# The Impact of Electroacupuncture Early Intervention on the Brain Lipidome in a Mouse Model of Post-traumatic Stress Disorder

**DOI:** 10.3389/fnmol.2022.812479

**Published:** 2022-02-10

**Authors:** Cui-Hong Zhou, Fen Xue, Qing-Qing Shi, Shan-Shan Xue, Tian Zhang, Xin-Xu Ma, Li-Sheng Yu, Chuang Liu, Hua-Ning Wang, Zheng-Wu Peng

**Affiliations:** ^1^Department of Psychiatry, Xijing Hospital, Air Force Medical University, Xi’an, China; ^2^Department of Toxicology, Shaanxi Key Lab of Free Radical Biology and Medicine, The Ministry of Education Key Lab of Hazard Assessment and Control in Special Operational Environment, School of Public Health, Air Force Medical University, Xi’an, China; ^3^Department of General Medicine, Shaanxi Provincial People’s Hospital, Xi’an, China; ^4^Department of Obstetrics, Xijing Hospital, Fourth Military Medical University, Xi’an, China

**Keywords:** electroacupuncture, post-traumatic stress disorder, lipidomics, hippocampus, prefrontal cortex, mouse model

## Abstract

The neuroprotective effect of electroacupuncture (EA) treatment has been well studied; growing evidence suggests that changes in lipid composition may be involved in the pathogenesis of post-traumatic stress disorder (PTSD) and may be a target for treatment. However, the influence of early EA intervention on brain lipid composition in patients with PTSD has never been investigated. Using a modified single prolonged stress (mSPS) model in mice, we assessed the anti-PTSD-like effects of early intervention using EA and evaluated changes in lipid composition in the hippocampus and prefrontal cortex (PFC) using a mass spectrometry-based lipidomic approach. mSPS induced changes in lipid composition in the hippocampus, notably in the content of sphingolipids, glycerolipids, and fatty acyls. These lipid changes were more robust than those observed in the PFC. Early intervention with EA after mSPS ameliorated PTSD-like behaviors and partly normalized mSPS-induced lipid changes, notably in the hippocampus. Cumulatively, our data suggest that EA may reverse mSPS-induced PTSD-like behaviors due to region-specific regulation of the brain lipidome, providing new insights into the therapeutic mechanism of EA.

## Introduction

Post-traumatic stress disorder is a stressor-related disorder characterized by avoidance, re-experiencing of trauma, and hyperarousal symptoms, and affects approximately 7–10% of the general population (Steenkamp et al., 2015; Careaga et al., 2016; O’Donovan et al., 2017). It is worth noting that the prevalence of PTSD increased significantly after the start of the COVID-19 pandemic (Guessoum et al., 2020; Mazza et al., 2020). PTSD is often associated with other psychiatric symptoms and functional impairment, such as depression, sleep disorders, anorexia as well as an increased risk of dementia and vascular neurodegenerative diseases (Song et al., 2020; Trottier et al., 2021). The disorder may persist over the patient’s lifetime and entail costs higher than those associated with other anxiety disorders (Brenner et al., 2019; Nichter et al., 2019; Tang et al., 2020). Currently, the main treatment strategy for PTSD is psychological intervention combined with pharmacotherapy. Although psychological intervention has strong support for improving fear extinction, emotion regulating and reducing distress responses in PTSD, psychological intervention is not effective for all patients, and its effects are prone to the spontaneous renewal of symptoms (Mayo et al., 2020; Weber et al., 2021). Moreover, selective serotonin reuptake inhibitors (SSRIs), such as paroxetine and sertraline are the first-line pharmacotherapy that has been shown to relieve PTSD symptoms including sleep problems, nightmares, anxiety and depression. However, these agents rarely induce remission and their benefits decline over time and might carry a risk of withdrawal syndromes and relapse (Mello et al., 2013; Shalev et al., 2017; Sbarski and Akirav, 2020). Therefore, investigation of the pathogenesis of PTSD and the development of new approaches for treating it are thus urgently needed (Krystal et al., 2017; Fenster et al., 2018).

Electroacupuncture is a modified form of traditional acupuncture. The latter is a technique of traditional Chinese medicine typically used in the field of complementary and alternative medicine (Langevin et al., 2011). Previous studies have mainly focused on the analgesic effect of EA (Zhang et al., 2014). However, multiple studies found that EA not only has neuromodulatory effects (Liu Z. et al., 2017; Chen et al., 2018), but can also ameliorate the symptoms of several neuropsychiatric diseases, such as the cognitive impairment in Alzheimer’s and Parkinson’s disease, and anxiety and depression in unmarried patients with polycystic ovarian syndrome (Li et al., 2019; Tamtaji et al., 2019; Wang et al., 2019; Zheng et al., 2021). Interestingly, a large number of studies reported that EA exerts neuroprotective effects by regulating the structure and function of the hippocampus and PFC (He et al., 2018; Chen et al., 2019; Marcus et al., 2020). For example, preclinical studies reported that EA ameliorate learning, memory and emotion regulating ability via regulating the neurochemical metabolism, cannabinoid receptors expression as well as activation of PV interneurons in the hippocampus and PFC (He et al., 2018; Chen et al., 2019; Marcus et al., 2020; Nie et al., 2020), which are regions of central importance in PTSD due to their prominent role in the neuroendocrine stress response and memory (Heyn and Herringa, 2019; Lambert and McLaughlin, 2019; Smith et al., 2019). Clinical and preclinical studies have previously reported that EA is protective against PTSD (Wang et al., 2012; Li et al., 2020). Moreover, clinical studies further found that EA improved fear extinction, ameliorated anxiety and insomnia in earthquake-related PTSD patients effectively (Hong and Efferth, 2016; Moiraghi et al., 2019). Likewise, a preclinical study also found that EA improved social avoidance and anxiety-like behaviors by reducing Lipocalin-2 expression and astrocyte activation in the hippocampus (Chen et al., 2021). And our previous work further found that early intervention or pretreatment with EA could enhance hippocampal neurogenesis and prevent PTSD-like behaviors in a rat model of PTSD through the Keap1/Nrf2 antioxidant signaling and endocannabinoid signaling (Xue et al., 2019; Zhou et al., 2019). Taken together, these results show that EA treatment is potentially therapeutic in cases of PTSD.

Lipids play an important role in maintaining and regulating brain development and function. Such functions include learning/memory and emotional behavior (Hashimoto et al., 2015; McDougall et al., 2017; Hussain et al., 2019). The involvement of lipids in modulating synaptic physiology, receptor pharmacology, energy generation, and brain metabolism has largely been established (Egawa et al., 2016; Araque et al., 2017; Wu et al., 2018). Recently, lipidomic alterations have been reported in psychiatric disorders, including bipolar disorder (Scola et al., 2016; Bartoli et al., 2017), schizophrenia (Kavoor et al., 2017), and anxiety disorder (Muller et al., 2015). Peripheral lipids may also be promising biomarker candidates to assist in the differential diagnosis of mild traumatic brain injury and PTSD (Huguenard et al., 2020) whereas EA was involved in regulating plasma and liver lipids metabolism in rodents (Li et al., 2018; Jung et al., 2021). However, to our knowledge, the changes in the brain lipidome occurring in PTSD and the link between the neuroprotective effects of EA and lipidomic alterations remain elusive.

Considering the above, we aimed to determine the lipid changes characteristic of PTSD and EA treatment, and the influence of early EA intervention on the lipidomic composition of the hippocampus and PFC in a mouse model of mSPS, which can produce robust symptoms and enhanced conditioned and sensitized fear responses that mimic PTSD (Wang et al., 2008).

## Materials and Methods

### Animals

Adult male C57BL/6 mice (8 weeks old, weighing 18–22 g) were obtained from the Fourth Military Medical University Animal Center (Xi’an, China). The mice were group-housed at four per cage in wire-bottomed cages at 20–25°C and maintained on a 12 h light/12 h dark daily cycle (lights on from 8:00 AM to 8:00 PM). Food and water were available *ad libitum* and all experiments were conducted during the light phase. The experimental procedures of this study were in accordance with the National Institutes of Health Guide for the Care and Use of Laboratory Animals and were approved by the Animal Use and Protection Committee of the Fourth Military Medical University.

### Experimental Design

**FIGURE 1:**
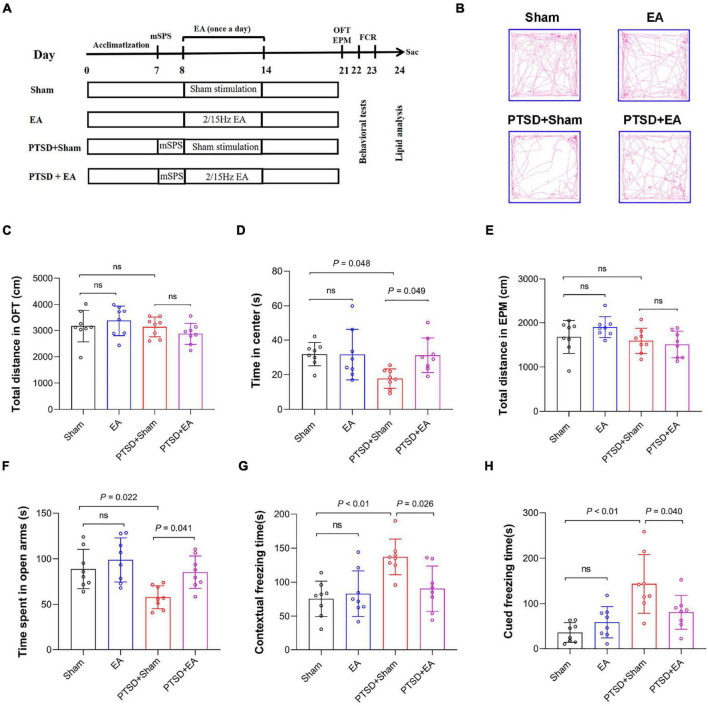
Early intervention using electroacupuncture (EA) ameliorates post-traumatic stress disorder (PTSD)-like behaviors in model mice treated with a modified single prolonged stress (mSPS) procedure. **(A)** The experimental design. After 1 week of adaptation, animals were administered mSPS or a sham treatment, then EA (2/15 Hz, 1.0 mA) or false stimulation was administered once a day for 30 min per day from days 8 to 14. Behavioral alterations were assessed from days 21 to 23, then mice were sacrificed (sac) for tissue collection. **(B)** Representative real-time movement traces in the open field test (OFT) for each group. **(C)** Quantification of the total distance traveled during the OFT. **(D)** Time spent in the center squares in the OFT. **(E)** Quantification of the total distance traveled in the elevated-plus maze test (EPMT). **(F)** Time spent in the open arms in the EPMT. **(G)** Freezing time measured in the contextual fear response test. **(H)** Freezing time measured in the cued fear response test. ns, no significant difference.

As shown in Figure 1A, after 7 days of acclimatization, 32 mice were randomly assigned to the following four groups (*n* = 8/group): Sham, EA, PTSD+Sham, and PTSD+EA. Mice in the PTSD+Sham and PTSD+EA groups were subjected to the mSPS protocol. Subsequently, mice in the EA and PTSD+EA groups were stimulated by EA at Baihui (GV20, Governing vessel meridian 20) with a frequency of 2/15 Hz and an intensity of 1 mA for 30 min each day, for seven consecutive days. Mice in the Sham and PTSD+Sham groups were administered Sham stimulation (EA-Sham treatment without application of electric current) for 30 min each day for seven consecutive days, and then handled in the home cage for 1 week. The researchers performing the behavioral testing were blinded to the animals’ group allocations, and the behavioral tests were performed 1 week after the final intervention. Subsequently, the mice were sacrificed and the PFC and hippocampus were immediately collected in liquid nitrogen for later lipidomic analyses by HPLC–MS/MS.

### Modified Single Prolonged Stress

The modified SPS paradigm was performed as described in a previous study (Feng et al., 2020), with some modifications. Briefly, mice were restrained for 2 h, followed by exposure to forced swimming for 20 min in an acrylic cylindrical tank (10 cm diameter, 25 cm height, water temperature 20–24°C) filled approximately two-thirds with water. Following gentle drying, the mice were subsequently exposed to diethyl ether inhalation until they lost consciousness for 2–3 min. After a 15-min recovery, the mice were exposed to a single electric foot shock (0.75 mA for 2 s) in a square box (360 mm width × 360 mm height × 360 mm depth) with a floor of stainless-steel rods and walls of aluminum and acrylic. Subsequently, the mice were returned to their home cages.

### Electroacupuncture Treatment

The electroacupuncture treatment was performed 24 h after the mSPS according to previous studies, with some modifications (Zheng et al., 2019; Geng et al., 2020; Chen et al., 2021). Briefly, mice were positioned in the induction case (a rectangular observation box) with 2.0 × MAC (minimum alveolar concentration, MAC) isoflurane for the initial 3 min for deep anesthetized condition, and then they were moved to the EA operating platform and maintained with 1.5 MAC isoflurane via nose mask. The disposable stainless-steel acupuncture needle was inserted into the murine equivalent of the acupoints Baihui (GV20, Governing vessel meridian 20) for 30 min once a day for seven successive days. Meanwhile, mice in sham groups were anesthetized with isoflurane and stimulated at the same acupoint without electricity for 30 min. The acupoint “Baihui (GV20, Governing vessel meridian 20)” is located at the intersection of the sagittal midline and the line linking the ears. To accomplish this, acupuncture needle (Shuzhou, Shuhzou Medical Appliance Factory Co., Ltd.; 0.30 × 25 mm) was inserted to a depth of approximately 2 mm, after which they were stimulated at a frequency of 2/15 Hz and an intensity of 1 mA (waveform: dilatational wave) with a G6805–2 electric acupuncture apparatus (No. 227033; Qingdao Xinsheng Ltd.) for 30 min. Meanwhile, another electrode was nipped to the tail to the formation of current loop.

### Behavioral Testing

Behavioral testing occurred 14 days after mSPS (Xi et al., 2021; Figure 1A). Animals were acclimated to a separate experimental room for at least 30 min prior to each test. The OFT was conducted prior to the elevated plus maze test on the same day, while the fear conditioning test was performed 24 h after the elevated plus maze test. All experiments were conducted under low light conditions to minimize anxiety effects and the test area was cleaned with 75% ethanol between tests.

#### Open Field Test

Based on a previous study (Sullivan et al., 2003), the OFT was performed in an unfamiliar soundproof box (50 × 50 × 50 cm) made of white polycarbonate. The floor had black grid marks dividing the behavioral arena into 36 squares comprising 16 central and 20 peripheral squares. Animals were placed in the center of the open-field box and activity was recorded for a period of 5 min by a video camera positioned directly above the arena. The recordings were analyzed by the open-field activity software Top Scan (Clever Sys Inc., Reston, VA, United States). The time spent and distance traveled in the central squares and entire arena were measured.

#### Elevated-Plus Maze Test

The EPMT was used to detecting anxiety-like behavior in mice (Liu et al., 2020). The maze apparatus (Dig Behav, Ji liang Co. Ltd., Shanghai, China) consisted of two opposing open arms of 35 × 6 cm and two opposing enclosed arms of 35 × 6 cm, all elevated 50 cm above the floor. During a test, a mouse was placed in the center square of the maze facing an open arm and its behavior recorded for 5 min by an automatic analyzing system (Top Scan, Clever Sys Inc., Reston, VA, United States). The number of arm entries and the time spent in open arms were used as indices of anxiety.

#### Fear Conditioning Response Test

Conditioned fear behavior was examined by the FCR test as described in previous reports (Feng et al., 2020). The experiments were performed in two dissimilar chambers: a shock chamber (Context A: a rectangular box with floors made of stainless-steel rods and walls of aluminum and acrylic) and a neutral-context chamber (Context B: a rectangular box with a white acrylic floor and an acrylic frame roof). The contextual fear conditioning paradigm and the cued fear conditioning paradigm each consisted of one training and two test sessions. For training, mice were acclimated to the shock chamber (Context A) for 180 s, then presented with a pure tone (28 s, 1 kHz, 80 dB) immediately followed by a foot shock (2 s, 0.75 mA). The tone–foot-shock pairing was delivered twice followed by a 90-s rest period, also in the chamber. Twenty-four hours later, mice were placed in the contextual chamber (Context A) for 5 min, without exposure to tone or foot shock, for the contextual fear response test. The cued fear response test was performed 1 h later. In this test, mice were acclimated to the neutral chamber (Context B) for 3 min. Subsequently, a neutral tone (4 kHz, 80 dB) was presented without foot-shock for 3 min. Mice were returned to the home cage after resting in the neutral chamber (Context B) for another 60 s. Freezing behavior was recorded and analyzed using a computerized automatic analysis system (Freezing Scan, Clever Sys Inc., Reston, VA, United States).

### Tissue Collection

After the behavioral test, all the mice were euthanized by cervical dislocation. Then the brains were subsequently removed and the PFC and hippocampi were dissected on ice immediately, then precisely weighed, frozen in liquid nitrogen, and stored at −80°C until lipidomic analysis were performed by high performance liquid chromatography-mass spectrometry (HPLC-MS).

### Sample Extraction

The Lipidome quantification and data analysis were performed as previously described (Cajka et al., 2017) and supported by Shanghai Applied Protein Technology Co., Ltd. Lipids were extracted with MTBE as described in a previous study (Jiang et al., 2017). Briefly, 30 mg samples were spiked with appropriate amounts of internal lipid standards (13 isotope mixtures internal lipid standards, SPLASH^®^ LIPIDOMIX MASS SPRC STANDARD, AVANTI,330707-1EA) and then homogenized with 200 μL of water and 240 μL of methanol. Subsequently, 800 μL of MTBE were added, and samples were ultrasonicated for 20 min at 4°C, then incubated at 25°C for 30 min. To separate the organic components, the solution was centrifuged at 10°C for 15 min at 14,000 × *g*. The upper layer, which was the organic solvent phase, was collected and the solvent evaporated under nitrogen. The solutes were stored at −80°C until use.

### Lipid Analysis by High Performance Liquid Chromatography–Tandem Mass Spectrometry

Reverse-phase chromatography was selected for ultra-HPLC separation using a CSH C18 column (ACQUITY UPLC CSH C18, 1.7 μm, 2.1 × 100 mm, Waters). Column temperature 45°C, Flow rate 300 μL/min. The lipid extracts were re-dissolved in 200 μL of 90% isopropanol/acetonitrile and centrifuged at 14,000 × *g* for 15 min; finally, 3 μL of each sample was injected for analysis. Solvent A was acetonitrile–water (6:4, v/v) containing 0.1% formic acid and 0.1 mM ammonium formate; solvent B was acetonitrile–isopropanol (1:9, v/v) also containing 0.1% formic acid and 0.1 mM ammonium formate. The initial mobile phase was 30% solvent B at a flow rate of 300 μL/min, which was held for two min, then linearly increased to 100% solvent B over 23 min, followed by equilibration in 5% solvent B for 10 min. The autosampler was maintained at 10°C. To avoid bias due to instrumentation errors, multiple samples were analyzed concurrently and signal fluctuations were detected in random order, even during sample analysis. Sampling of the queue was performed every eight samples using one of the quality control (QC) samples to monitor the stability of the analysis and evaluate the reliability of the experimental data.

After separation by ultra-HPLC, samples were analyzed using a Q Exactive™ plus mass spectrometer (Thermo Fisher Scientific, Waltham, MA, United States) using the following parameters. For positive-ion mode: heater temperature, 300°C; sheath gas flow rate, 45 arbitrary units (arb); auxiliary gas flow rate, 15 arb; sweep gas flow rate, 1 arb; spray voltage, 3.0 kV; capillary temperature, 350°C; S-Lens radio frequency (RF) level, 50%; and MS1 scan range, 200–1800 m/z. For negative-ion mode: heater temperature, 300°C; sheath gas flow rate, 45 arb; auxiliary gas flow rate, 15 arb; sweep gas flow rate, 1 arb; spray voltage, 2.5 kV; capillary temperature, 350°C; S-Lens RF level, 60%; and MS2 scan range, 250–1800 m/z.

### Lipid Identification Using LipidSearch™

LipidSearch (Thermo Fisher Scientific, Waltham, MA, United States) is a search engine used to identify lipid species based on MS/MS data. LipidSearch contains data on >30 lipid classes and >1,500,000 ion fragments. The mass tolerances for both molecular precursors and fragment ions were set to 5 ppm. The displayed product ion threshold was set at five, and grades A, B, C, D were all used in the identification (ID) quality filtering. All lipid classes in the database, including 71 sub-species, were chosen for identification. Adducts of H^+^ and NH_4_^+^ were selected for positive-mode searches, and adducts of H^–^ and CH_3_COO^–^ were selected for negative-mode searches, since ammonium acetate was used in the mobile phases.

### Statistical Analysis

Statistical analyses were performed using SPSS v.19.0 software (SPSS Inc., Chicago, IL, United States). The results of behavioral testing and the characterizations of lipid compositions are presented as mean ± standard deviation (SD), and were evaluated by one- or two-way analysis of variance (ANOVA) and corrected by Bonferroni *post-hoc* test for pairwise comparisons. A *P*-value <0.05 was deemed statistically significant. The correlations between lipid species levels and behaviors were analyzed using the Pearson correlation.

The raw lipidomic data were processed using LipidSearch software for peak recognition, lipid-peak extraction (secondary appraisal), peak alignment, and quantitative processing. After normalizing to the total peak intensity and integration using the Perato scaling method, the processed data were imported into SIMPCA-P 14.1 (Umetrics, Umea, Sweden) for multivariate statistical analysis, which included principal component analysis, partial least squares discriminant analysis, and orthogonal partial least squares discriminant analysis. Lipids with significant differences were identified based on a combination of (1) statistically significant thresholds of variable influence on projection values (obtained from orthogonal partial least squares discriminant analysis and two-tailed Student’s *t*-tests) and (2) mapping of volcano, hierarchical-cluster, and correlation analyses using R software (Xue et al., 2020).

## Results

### Electroacupuncture Treatment Ameliorates Post-traumatic Stress Disorder-Like Behaviors in Modified Single Prolonged Stress-Treated Mice

First, we determined the effect of EA treatment on PTSD-like behaviors (Figure 1A). Two-way ANOVA revealed that mSPS and EA treatment did not induce any motor impairment in mice because in the OFT, we observed no significant differences in the total distance traveled in either the mSPS or the EA treatment factors Figures 1B,C). However, significant differences were observed in the time spent in the center squares of the OFT, time spent in the open arms of the EPMT, as well as freezing time in both contextual fear and cued fear test between the four groups (Figures 1D–H and Supplementary Table S1). *Post hoc* comparisons further showed that mSPS markedly reduced the time spent in the center in the OFT and the time spent in the open arms in the EPMT (PTSD+Sham vs. Sham, *P* < 0.05), and that these measures were ameliorated after EA treatment (PTSD+EA vs. PTSD+Sham, *P* < 0.05). Additionally, the PTSD+Sham group showed a significant increase in freezing time in both contextual fear and cued fear conditioning tests when compared with the Sham group (*P* < 0.01). Moreover, EA treatment significantly decreased freezing time and alleviated fear-like behaviors in the mSPS-treated mice (PTSD+EA vs. PTSD+Sham, *P* < 0.05, Figures 1G,H). These results suggest that EA treatment can ameliorate anxiety PTSD-like behaviors in mSPS-treated mice.

### Identification of the Number of Lipid Compounds

According to the International Lipid Classification and Nomenclature Committee, lipid compounds are divided into eight types. Each class type can be divided into different subtypes with polarity as the head of the class (lipid class). To achieve a three-level classification of lipid compounds, each subgroup was divided into different molecular species (lipid species) based on the different saturabilities or lengths of the carbon chain. We identified 1,048 lipid species and 29 lipid classes in the samples of each group (Supplementary Figure S1).

### Electroacupuncture Treatment Effect: Hippocampal Lipid Classes

**FIGURE 2:**
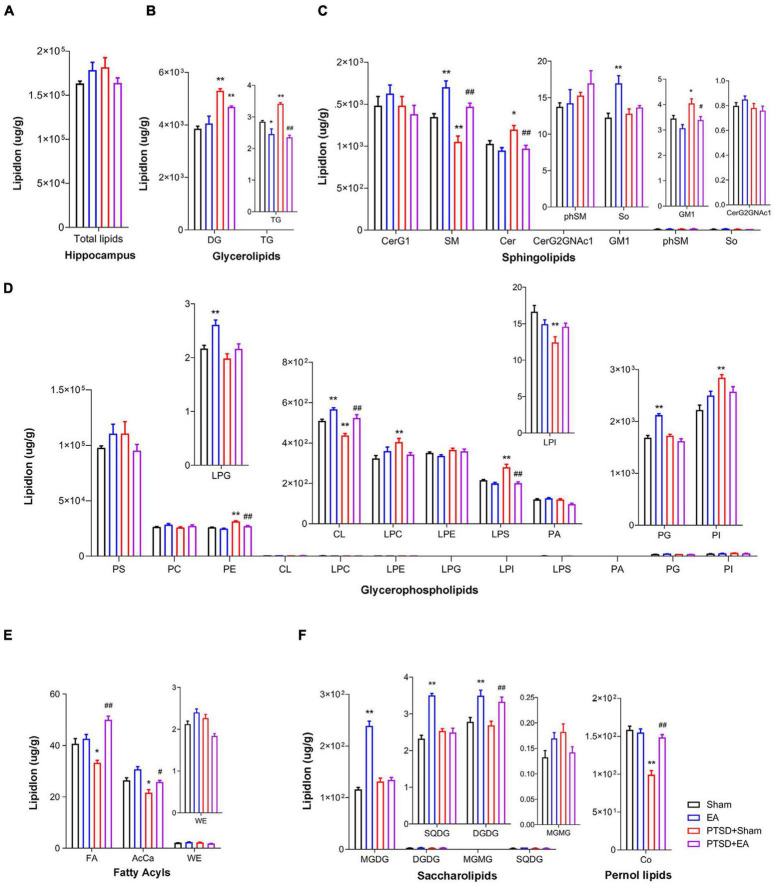
Groupwise alterations in the lipidomic profiles in the hippocampus. **(A)** Total lipid concentration, **(B)** glycerolipids, **(C)** sphingolipids, **(D)** glycerophospholipids, **(E)** fatty acyls, **(F)** saccharolipids and prenol lipids. DG, diglyceride; TG, triglyceride; CerG, glucosylceramides; SM, sphingomyelin; Cer, ceramides; GM1, gangliosides; phSM, phytosphingosine sphingomyelin; So, sphingosine; PS, phosphatidylserine; PC, phosphatidylcholine; PE, phosphatidylethanolamine; CL, cardiolipin; LPC, lysophosphatidylcholine; LPE, lysophosphatidylethanolamine; LPG, lysophosphatidylglycerol; LPI, lysophosphatidylinositol; LPS, lysophosphatidylserine; PA, phosphatidic acid; PG, phosphatidylglycerol; PI, phosphatidylinositol; FA, fatty acid; AcCa, acylcarnitine; WE, wax esters; MGDG, monogalactosyldiacylglycerol; DGDG, digalactosyldiacylglycerol; MGMG, monogalactosylmonoacylglycerol; SQDG, sulfoquinovosyldiacylglycerol; Co, coenzyme; PTSD, post-traumatic stress disorder; EA, electroacupuncture; *P < 0.05 vs. Sham; **P < 0.01 vs. Sham; ^#^*P* < 0.05 vs. PTSD; ^##^*P* < 0.01 vs. PTSD+Sham.

As shown in Figure 2, we found no statistical intergroup differences in total lipid levels (*F_3_,_28_* = 1.598, *P* = 0.2122) in the hippocampus (Figure 2A). However, in this structure, we observed that concentrations of 17 lipids were affected by mSPS factor and 18 lipids were affected by EA factor in the hippocampus (Figures 2B–F and Supplementary Table S2). *Post hoc* comparisons revealed that mice in the PTSD group exhibited significantly elevated levels of DG, TG, Cer, GM1, LPS, LPC, PI, and PE but reduced levels of SM, CL, LPI, FA, AcCa, and Co (PTSD+Sham vs. Sham, *P* < 0.05). EA treatment effectively decreased the levels of Cer, GM1, LPS, PE, and TG and increased the levels of SM, CL, AcCa, FA, and Co (PTSD+EA vs. PTSD+Sham, *P* < 0.05). However, the changes in DG, LPI, LPC, and PI induced by mSPS in the hippocampus were not normalized by EA treatment.

**FIGURE 3:**
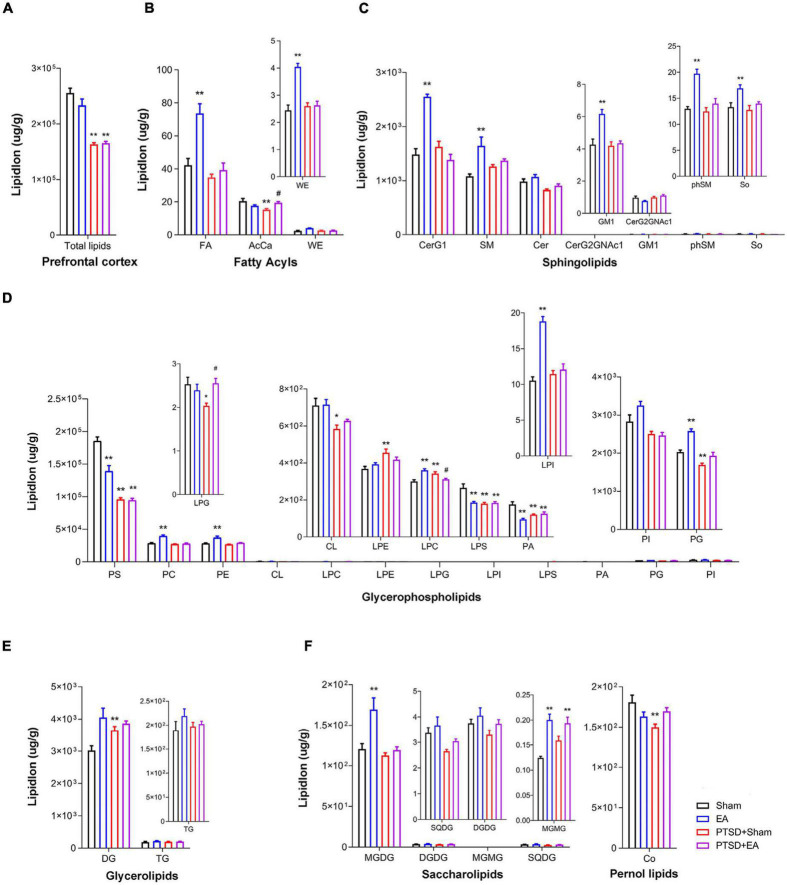
Analysis of correlations between PTSD-like behaviors and lipid levels in **(A)** the hippocampus and **(B)** the PFC, using Pearson’s correlation coefficient. *P < 0.05; **P < 0.01.

Correlation analysis showed that the time spent in the center squares in the OFT was positively correlated with levels of AcCa, CL, LPI, SM, Co, and DGDG but negatively correlated with levels of TG, LPS, and PE. The time spent in the open arms in the EPMT was positively correlated with levels of FA, CL, LPG, LPI, PC, SM, Co, and DGDG but negatively correlated with levels of DG, TG, LPC, LPS, PE, Cer, and GM1. Moreover, the contextual freezing time recorded in the FCR test was positively correlated with levels of DG, LPC, LPS, PE, and PI but negatively correlated with levels of AcCa, FA, CL, LPG, LPI, SM, and Co. The cued FCR freezing time was positively correlated with levels of DG, LPC, LPS, PE, PI, and Cer but negatively correlated with levels of AcCa, FA, CL, LPI, and Co (Figure 3A and Supplementary Table S3). These results suggest that hippocampal levels of AcCa, FA, CL, LPI, SM, and Co are negatively correlated with the severity of PTSD, while levels of DG, LPC, PE, LPS, and Cer are positively correlated with severity.

### Electroacupuncture Treatment Effect: Prefrontal Cortex Lipid Classes

**FIGURE 4:**
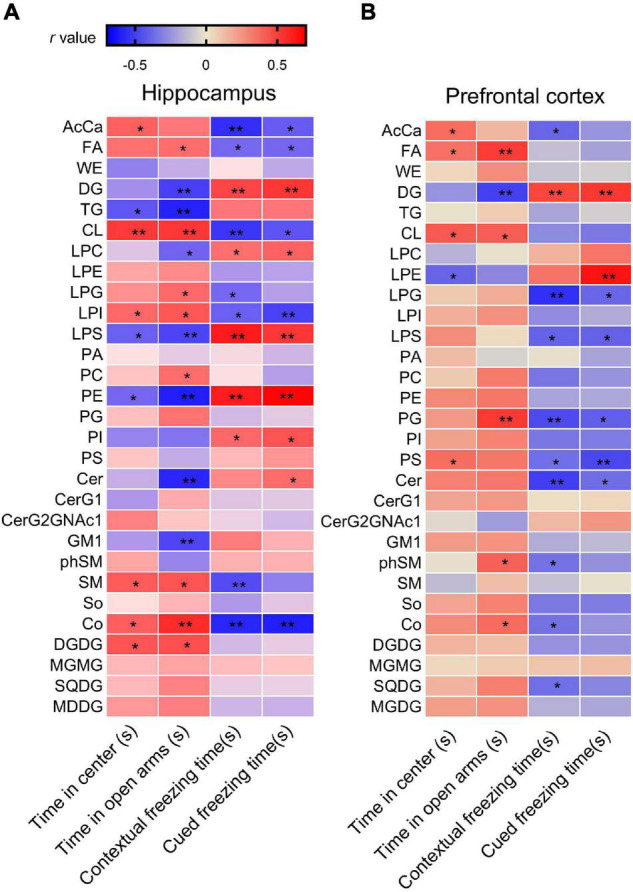
Groupwise alterations in the lipidomic profiles in the prefrontal cortex (PFC). **(A)** Total lipid concentration, **(B)** fatty acyls, **(C)** sphingolipids, **(D)** glycerophospholipids, **(E)** glycerolipids, **(F)** saccharolipids and prenol lipids. FA, fatty acid; AcCa, acylcarnitine; WE, wax esters; CerG, glucosylceramides; SM, sphingomyelin; Cer, ceramides; GM1, gangliosides; phSM, phytosphingosine sphingomyelin; So, sphingosine; PS, phosphatidylserine; PC, phosphatidylcholine; PE, phosphatidylethanolamine; CL, cardiolipin; LPC, lysophosphatidylcholine; LPE, lysophosphatidylethanolamine; LPG, lysophosphatidylglycerol; LPI, lysophosphatidylinositol; LPS, lysophosphatidylserine; PA, phosphatidic acid; PG, phosphatidylglycerol; PI, phosphatidylinositol; DG, diglyceride; TG, triglyceride; MGDG, monogalactosyldiacylglycerol; DGDG, digalactosyldiacylglycerol; MGMG, monogalactosylmonoacylglycerol; SQDG, sulfoquinovosyldiacylglycerol; Co, coenzyme; PTSD, post-traumatic stress disorder; EA, electroacupuncture; *P < 0.05 vs. Sham; **P < 0.01 vs. Sham; ^#^*P* < 0.05 vs. PTSD+Sham.

As shown in Figure 4, we observed significant intergroup differences in total lipid levels in the PFC (*F*_3,28_ = 39.91, *P* < 0.001, Figure 4A); mSPS treatment decreased the total lipid concentration in this region (PTSD+Sham vs. Sham, *P* < 0.01). Moreover, we found that concentrations of 19 lipids were affected by mSPS factor and 17 lipids were affected by EA factor in the PFC (Figures 4B–F and Supplementary Table S2). *Post hoc* comparisons further revealed that mice in the PTSD+Sham group exhibited significantly decreased levels of AcCa, PS, LPG, CL, LPS, PA, PG, and Co and increased levels of LPE, LPC, and DG (PTSD+Sham vs. Sham, *P* < 0.05). EA treatment increased the levels of AcCa and LPG (PTSD+EA vs. PTSD+Sham, *P* < 0.05). Although the changes in PS, CL, LPS, PA, PG, Co, LPE, and DG induced by mSPS in the PFC were not normalized by EA treatment, other changes were, and we found no significant differences in the levels of LPE, CL, PG, and Co between PTSD+EA and Sham.

Correlation analysis showed that the time spent in the center squares in the OFT was positively correlated with levels of AcCa, FA, CL, and PS in the PFC, but negatively correlated with the level of LPE. The time spent in the open arms of the EPMT was positively correlated with levels of FA, CL, PG, phSM, and Co but negatively correlated with the level of DG. The contextual freezing time in FCR was positively correlated with the level of DG but negatively correlated with the levels of AcCa, LPG, LPS, PG, PS, Cer, phSM, and SQDG. The cued freezing time in the FCR was positively correlated with DG and LPE but negatively correlated with levels of LPG, LPS, PG, PS, and Cer (Figure 3B and Supplementary Table S3). These results suggest that the levels of PS and PG in the PFC negatively correlate with the severity of PTSD, and the level of DG positively correlates with severity.

### Electroacupuncture Treatment Effect: Fatty Acid Composition

**FIGURE 5:**
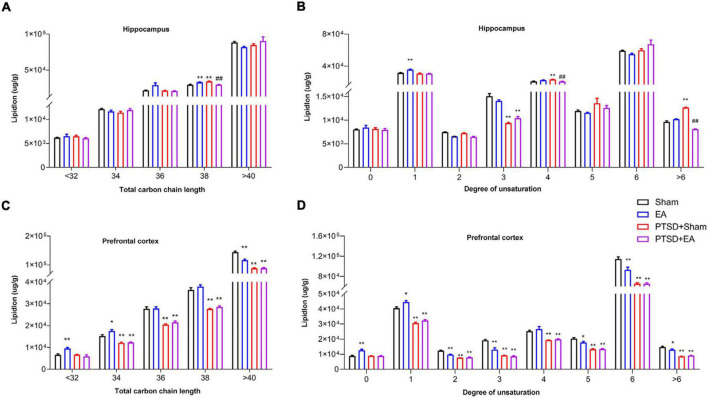
Groupwise alterations in fatty acid composition in the hippocampus and PFC. Results of analysis of fatty acyl composition by **(A,C)** chain length (number of carbons) totals and **(B,D)** degree of unsaturation totals. PTSD, post-traumatic stress disorder; EA, electroacupuncture; *P < 0.05 vs. Sham; **P < 0.01 vs. Sham; ^##^*P* < 0.01 vs. PTSD+Sham.

As shown in Figure 5, mSPS treatment led to a significant alteration in the fatty acyl chain profile of lipids in the hippocampus and PFC. In the hippocampus, in the PTSD+Sham group, levels of long-chain fatty acyls with 38 carbons (38C, Figure 5A) and levels of polyunsaturated fatty acyls with 4 double bonds and >6 double bonds were increased, while levels of polyunsaturated fatty acyls with 3 double bonds were decreased (Figure 5B). Notably, these changes were ameliorated by EA treatment. On the other hand, in the PFC, levels of long-chain fatty acyls with >32 carbons (38C) and levels of total unsaturated fatty acids were decreased in the PTSD+Sham group, and these changes were not attenuated after EA treatment (Figures 5C,D).

### Characteristic Lipid Species in the Brain and Their Correlation With Post-traumatic Stress Disorder-Like Behaviors

Lipidomic profiling at the species level revealed additional intergroup changes in the concentrations of lipids in the hippocampus and PFC. According to the characteristics of the PLS-DA model, the data of lipids in the hippocampus and PFC could be well distinguished between the four groups (Supplementary Figures S2A,D). The differential lipids were determined based on the following criteria: (1) FC > 1.5 or <0.067 and (2) *P-*value < 0.05. A total of 241 lipid species were dysregulated after mSPS and 42 of them were normalized after EA treatment in the hippocampus (Supplementary Figures S2B,C). A total of 130 lipid species were dysregulated after mSPS and 11 of them were normalized after EA treatment in the PFC (Supplementary Figures S2E,F).

**FIGURE 6:**
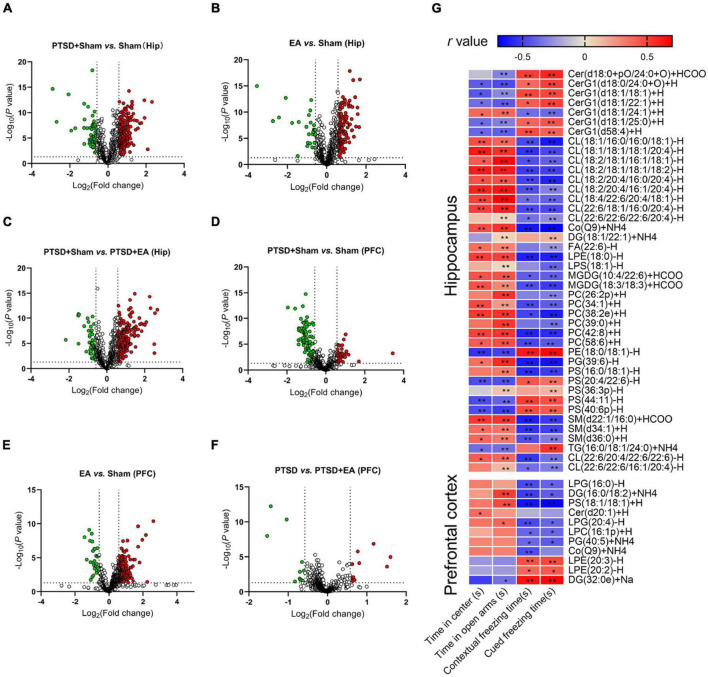
Groupwise characterization of lipid species and their correlations with PTSD-like behaviors. **(A–C)** Volcano map showing decreased (green dots) and increased (red dots) lipid species comparing the PTSD+Sham group and Sham, the EA group and Sham, and the PTSD+Sham group and the PTSD+EA group in the hippocampus. **(D–F)** Same comparisons for the PFC. The vertical dotted lines in the graph means Log_2_(0.667) and Log_2_(1.5), the horizontal dotted line means –Log_10_(0.05). **(G)** Correlation between PTSD-like behaviors and levels of lipid species in the hippocampus and PFC. The behavioral correlations were analyzed using Pearson’s correlation coefficient. Hip, hippocampus; PFC, prefrontal cortex; *P < 0.05; **P < 0.01.

As shown in Figures 6A–C and Supplementary Table S4, concentrations of 60 lipids such as PC(33:0)+H, DG(34:1e),+Na and CL(18:2/20:4/16:0/20:4)-H were decreased and those of 181 lipids such as TG(16:0/18:1/20:1)+NH4, PS(42:7p)-H, and TG(17:0/18:1/18:1)+NH4 were increased in the hippocampus in the PTSD+Sham group compared with the Sham group (Figure 6A). Concentrations of 24 lipids such as DG(34:1e)+Na, LPI(18:0)-H, and TG(18:1/18:1/18:3)+NH4 were decreased and those of 134 lipids such as PC(38:2)+H, PS(18:1/24:0)-H, and DG(18:1/22:1)+NH4 were increased in the hippocampus in the EA group compared with the Sham group (Figure 6B). EA treatment increased 47 lipids such as PC(18:0p/20:1)+HCOO, MGDG(10:4/22:6)+HCOO, and CerG1(d18:1/22:1)+H and decreased 192 lipids such as TG(18:1/18:1/18:1)+NH4, PS(42:7p)-H, and TG(17:0/18:1/18:1)+NH4 in the hippocampus in PTSD-treated mice (Figure 6C). Notably, 42 of the 241 lipid species in the hippocampus that were changed after PTSD treatment, such as Cer(d18:0+pO/24:0+O)+HCOO, PC(34:1)+H, and PS(20:4/22:6)-H, were normalized after EA treatment (see Supplementary Table S5 for details), suggesting that changes in these lipid species may be related to the biological effects of EA. Moreover, correlation analysis (Figure 6G) showed that the concentrations of nine lipids such as CerG1(d18:0/24:0+O)+H, PE(18:0/18:1)+H, and PS(44:11)-H in the hippocampus were negatively correlated with the time spent in the center squares in the OFT and in the open arms of the EPMT, but positively correlated with contextual and cued freezing time in the FCR. Concentrations of 23 lipids such as CL(18:1/16:0/16:0/18:1)-H, PC(34:1)+H, and SM(d34:1)+H in the hippocampus were positively correlated with the time spent in the center squares in the OFT and in the open arms in the EPMT, but negatively correlated with contextual and cued freezing time in the FCR (see Supplementary Table S6 for details), suggesting that changes in these lipid species in the hippocampus are related to the severity of PTSD.

Like the hippocampus, the PFC analysis revealed changes in the lipid concentrations in multiple species following mSPS and EA treatments (see Figures 6D–F and Supplementary Table S4). Generally, the levels of 93 lipids such as PS(18:0/22:4)-H, PS(22:6/22:6)-H, and PS(42:7p)-H were decreased, and the levels of 37 lipids such as PC(32:0)+H, WE(21:1)+NH4, and TG(16:0/20:4/20:4)+NH4 were increased in the PFC of PTSD-treated mice compared with Sham (Figure 6D). The concentrations of 30 lipids such as PS(38:4p)-H, PS(42:7p)-H, and PC(32:0)+H were decreased and those of 201 lipids such as PE(34:2p)-H, TG(18:3/18:2/18:2)+NH4, and PC(33:0)+H were increased in EA-treated mice compared with Sham (Figure 6E). Moreover, EA treatment increased the levels of 11 lipids such as LPG(16:0)-H, DG(16:0/18:2)+NH4, and PG(40:5)+NH4, and decreased the levels of 10 lipids such as PS(18:0/22:4)-H, LPE(20:2)-H, and LPE(20:3)-H compared with the PTSD+Sham group (Figure 6F). Notably, levels of 11 of the 130 lipid molecules in the PFC changed after mSPS such as LPG(16:0)-H, DG(16:0/18:2)+NH and LPE(20:3)-H were normalized after EA treatment (see Supplementary Table S5 for details), suggesting that changes in these species in the PFC may be related to the biological effects of EA. Correlation analysis further showed that concentrations of LPG(16:0), DG(16:0/18:2)+NH4, PS(18:1/18:1)+H, LPG(20:4)-H, LPC(16:1p)+H, and PG(40:5)+NH4 in the PFC were negatively correlated, and concentrations of LPE(20:3)-H, LPE(20:2)-H, and DG(32:0e)+Na were positively correlated with contextual and cued freezing time in the FCR test (see Supplementary Table S6 for details), suggesting that changes in these lipid species in the PFC are related to the severity of fear-like behaviors. Moreover, concentrations of DG(16:0/18:2)+NH4, PS(18:1/18:1)+H, and LPG(20:4)-H were positively correlated, and DG(32:0e)+Na was negatively correlated with the time spent in the open arms in the EPMT, suggesting that changes in these lipid species in the PFC are related to the severity of anxiety.

## Discussion

In the present study, we performed comprehensive lipid profiling based on HPLC–MS/MS to assess the impact of EA treatment on lipidomic changes in the hippocampus and PFC of mice exposed to mSPS. We found that mSPS induced remarkable anxiety-like behaviors, fear-learning defects, and lipid changes in the brain, specifically in glycerolipids, glycerophospholipids, and sphingolipids. Moreover, the changes in lipid levels were more extensive in the hippocampus than in the PFC. Notably, early EA intervention attenuated mSPS-induced abnormal behaviors and partly normalized mSPS-induced lipid changes, notably in the hippocampus. Thus, a region-specific dysfunction of the brain lipidome may be involved in the pathogenesis of PTSD, and a region-specific regulation of the brain lipidome might partly account for the therapeutic effects of EA.

In PTSD, the hippocampus and PFC are regions of central importance due to their prominent role in both the neuroendocrine stress response and memory alterations (Smith et al., 2019; Harnett et al., 2020). Atrophy of the hippocampus is one of the obvious pathologic changes in patients with PTSD and in animal models (Lindauer et al., 2006; Kikuchi et al., 2008). Hippocampal dysfunction might interact with traumatic experiences to influence the etiology and maintenance of PTSD (Acheson et al., 2012; Bae et al., 2020) whereas the amelioration of hippocampal structure and function was related to the alleviation of PTSD (Nie et al., 2014, 2021). In terms of PFC, a clinical study found that youths with PTSD had sustained decreases in gray matter volume in the right ventromedial PFC (vmPFC) and bilateral ventrolateral PFC as well as decreased ventrolateral PFC-hippocampus connectivity over time (Heyn et al., 2019). Consistent with these results, PTSD remission has been associated with expansion of frontal pole surface area and an increase in vmPFC thickness over time (Heyn and Herringa, 2019). Moreover, a preclinical study found that the protective effects of miR-132 downregulation against behavioral impairment in rats exposed to single prolonged stress was related to a reduction in apoptosis in the PFC and an upregulation of brain-derived neurotrophic factor (BDNF) (Tong et al., 2021). Notably, changes in the lipid composition of the hippocampus and PFC have been consistently associated with cognitive impairment and depressive-like behaviors (Oliveira et al., 2016; Xue et al., 2020; Zhou et al., 2021). On the other hand, the function of several lipids is related to apoptosis and neurogenesis (Huang and Freter, 2015; Knobloch, 2017; Oddi et al., 2020) and the effects of EA on the neurogenesis of hippocampus as well as its neuroprotective effect against neural damage have been largely declared (Kim et al., 2018; Xing et al., 2018; Pei et al., 2021). Therefore, we hypothesized that changes in the lipid composition of the hippocampus and PFC might also be associated with fear and anxiety-like behaviors in PTSD. However, the correlation between the regulatory effects of EA on brain lipidomics and the morphology of hippocampal and PFC is still unclear and needs further investigation.

Glycerophospholipids and sphingolipids are important membrane lipid components that allow for the construction of a physical barrier (Muller et al., 2015; Muallem et al., 2017) and play crucial roles in regulating cell signaling (Nadler et al., 2015). Research has gradually revealed a general role for these components in neuropsychiatric diseases. Levels of PC and Cer are changed in the brain tissue of patients with bipolar disorder (Schwarz et al., 2008), and the relative abundances of LPC, PE, and PI in the plasma were positively correlated with the severity of depression (Liu et al., 2016; Ogawa et al., 2020). Moreover, levels of Cer are elevated in patients with major depression and bipolar disorder (Brunkhorst-Kanaan et al., 2019), and concentrations of SM correlate with depression and anxiety symptoms (Demirkan et al., 2013). Consistent with these observations, we found that mSPS induced significant changes in glycerophospholipid and sphingolipid levels in both the hippocampus and PFC. In the PTSD group, significant increases in hippocampal levels of LPS, LPC, PI, PE, Cer, and GM1 were observed, and in PFC, significant increases in levels of LPE and LPC were observed; significant decreases were observed in hippocampal CL, LPI, and SM and in prefrontal PS, LPG, CL, LPS, PA, and PG. Notably, changes in SM, Cer, GM1, PE, CL, and LPS in the hippocampus, which were correlated with anxiety or cognitive impairment, were alleviated after EA treatment. However, in the PFC, only changes in LPG and LPC were alleviated after EA treatment. SM constitutes the vast majority of cellular sphingolipid, and can be hydrolyzed to Cer by acid sphingomyelinase (ASM). Accumulating evidences suggest that the ASM/Cer system could potentially be a novel antidepressant target, and increased activity of the ASM/Cer system might be both an effect and a cause of immune system-mediated inflammation and dysregulation of oxidative stress (Kornhuber et al., 2014; Kurz et al., 2019). GM1 is a key factor in maintaining the mammalian neuronal functions and avoiding neurodegeneration (Chiricozzi et al., 2020; Meng et al., 2020) and CL plays a role in the regulation of cellular energy metabolism and mitochondrial function (Acaz-Fonseca et al., 2020). LPC homeostasis is an endogenous mediator of myelin injury (Plemel et al., 2018); LPC levels are elevated in the cerebrospinal fluid of patients with multiple sclerosis (Shanta et al., 2012) and in the brain following ischemia (Pieragostino et al., 2015). LPS and LPG represent an immunomodulatory effect (Favila et al., 2015; Ogasawara et al., 2018). PE is involved in the modulation of membrane fluidity and properties that protect the cell under conditions of oxidative stress (Wallner et al., 2018). Thus, mSPS-induced anxiety and fear-like behaviors are related to an imbalance in levels of specific glycerophospholipids and sphingolipids in both the hippocampus and PFC. Lipidomic analyses revealed that 42 species, mainly belonging to the CL, PC, CerG1, PS, and SM series, were changed after mSPS in the hippocampus, while 11 species, mainly belong to the LPG and LPE series, were changed in the PFC, all of which were alleviated after EA. These results further indicate that the modulation of glycerophospholipid and sphingolipid levels after EA was brain area-specific, and that the lipid regulatory effect of EA was greater in the hippocampus than in the PFC.

In addition to the disturbance in levels of membrane lipid classes observed in the pathophysiology of PTSD, a growing body of evidence indicates that abnormalities in glycerolipid and fatty acyl compositions may also be involved (de Vries et al., 2016; Huguenard et al., 2020). The present study showed that mSPS increased the levels of glycerolipids in both the hippocampus (DG and TG) and the PFC (DG). However, only levels of TG, which were positively correlated with anxiety-like behaviors, were normalized after EA treatment, suggesting that the modulation of TG in the hippocampus is at least partially responsible for the protective effects of EA against PTSD.

In the brain, AcCa compounds functionally alter and stabilize membranes, thereby improving mitochondrial function and enhancing antioxidant activity (Rutkowsky et al., 2014; Tarasenko et al., 2018). Recent studies have demonstrated that AcCa levels in the plasma of depressed subjects are lower than those of healthy controls and that these alterations correlate with the severity of the patients’ depressive symptoms (Cassol et al., 2015). Our data show that mSPS induced a significant decrease in AcCa levels in both the hippocampus and PFC, which was negatively correlated with contextual freezing time and was normalized after EA treatment. Thus, a reduction in brain AcCa is involved in the pathophysiology of stress-induced fear-like behaviors and recovery of AcCa levels is associated with the protective effects of EA observed in this study.

The biophysical properties of lipids are influenced by the chain length (number of carbons) and the degree of saturation of the constituent fatty acyls (Guha et al., 2008). Modulation of the composition of the fatty acyl chains and degree of saturation of membrane lipids can potentially affect neuronal functioning, at least in part through altered function of membrane-bound proteins (Carta et al., 2014; Oliveira et al., 2016). The present study found that changes in the composition of fatty acyls in the hippocampus attributable to mSPS were ameliorated after EA treatment. However, the more extensive changes observed in the PFC were not normalized after EA treatment, suggesting that fatty acyl composition in the PFC is more vulnerable to mSPS and does not recover as quickly as that in the hippocampus. Notably, we also found that after mSPS, the concentration of Co(Q9) was lowered in both the hippocampus and PFC and was effectively normalized after EA treatment. CoQ is a redox-active molecule that plays a fundamental role in mitochondrial energy generation and functions as a potent endogenous antioxidant (Pandey et al., 2018). Our results indicate that a disturbance of the brain antioxidant system is involved in the pathogenesis of PTSD and that Co(Q9)+NH4 is potentially a molecular target for the treatment of PTSD.

Recent work has previously reported an influence of acupuncture treatment on both depressive-like behaviors and the lipidomics of the liver (Jung et al., 2021). However, the neuroprotective effects of EA are closely related to its action parameters and action time, and precedents exist for suspecting that early intervention using EA might be an effective strategy for preventing the development of PTSD symptoms (Peng et al., 2013; Nie et al., 2014). Baihui (GV20, Governing vessel meridian 20) is located above the apex auriculate at which the fractional amplitude of low-frequency fluctuation could fully spread to mood relevant hippocampus and PFC circuits (Mao et al., 2020) and is considered to be the optimized acupoints for mental illness (Lin et al., 2019). EA at GV20 could effectively ameliorate depressive-and anxiety-like behaviors in PTSD and depression models (Yue et al., 2018; Chen et al., 2021), as well as improve the synaptic plasticity and metabolism in the hippocampus (He et al., 2021). Previous studies found that EA stimulation (2/15 Hz) applied at the GV20 enhanced motor performance recovery and BDNF expression in rats with cerebral infarction (Kim et al., 2012). EA treatment has also been shown to reduce glutamate toxicity and exert antiapoptotic effects in a rat stroke model (Zhou et al., 2013; Zhu et al., 2013). Importantly, our previous work found that early EA intervention applied at the GV20 point with the same frequencies (2/15 Hz) prevents PTSD-like behaviors in a rat model of PTSD (Xue et al., 2019). Consistent with these results, the present study indicates that early EA intervention applied at the GV20 point with 2/15 Hz for seven continuous days exerts protective effects on experimental PTSD in mice and mobilizes changes in brain lipid compositions, mainly in the hippocampus. Furthermore, the effects of EA on brain metabolism have been reported both in preclinical and clinical studies (Yang et al., 2014; Liu W. et al., 2017; Hou et al., 2020) and EA directly influence lipidomics in both hippocampus and PFC. Therefore, we speculate that different frequencies and current intensity of EA might induce different neuromodulation effects by influence the composition of lipids in the brain and EA may directly inhibit the effect of stress on the brain by regulating metabolism. Neither did we investigate the effects of EA at different time points, nor the relationships between different parameters of EA and the composition of lipids in the brain, which should be the goals of future research.

Taken together, our results indicate that lipidomic changes in the PFC and hippocampus may be a key aspect of the pathogenesis of PTSD. Importantly, the functions of these lipids are mainly focused on anti-inflammation, energy metabolism, oxidative stress, and neuroprotection, which may partly explain the neuroprotective effect of EA. The observation of a correlation between levels of oxidative stress, inflammation, or levels of neurotrophic factors in the hippocampus or PFC might provide further insight into this hypothesis. The present study found that levels of AcCa, CL, and Co were decreased after EA while that of LPC was increased in both hippocampus and PFC. However, changes in LPS and DG showed an opposite trend in the hippocampus and PFC, which might relate to the divergent structures and functions of these brain regions. The precise mechanisms of brain area-specific modulation after EA and the effects of specific lipid species on PTSD-like behaviors require further investigation. Meanwhile, although all mice in this study were exposed to isoflurane inhalation anesthesia, we cannot rule out an anesthetic effect on our results due to the regulatory effect of isoflurane on brain lipidomics and its potential influence on brain oxidative stress, inflammation, and levels of neurotrophic factors (Lee et al., 2015; Miao et al., 2017; Zhou et al., 2017). Moreover, the biological function of brain lipids has not been fully clarified, especially the function of lipids at the species level is still largely unclear. This study made a correlation analysis between differential lipid and behavioral data, which is based on lipid content and behavioral score. Although a large number of lipid molecular levels have been found to be related to PTSD-like behavior, this association does not explain the biological and molecular mechanism behind it. Only by adjusting the target lipid molecules and seeing whether they affect PTSD-like behavior can we further screen which potential molecular targets were closely related to PTSD.

In summary, our study revealed distinct changes in lipid composition within the hippocampus and PFC in mice after exposure to mSPS. We further confirmed that the hippocampus is more sensitive to mSPS-induced lipid modulation than is the PFC and showed that EA is effective in normalizing the changes in lipid composition in the hippocampus, notably changes in sphingolipids and glycerophospholipids. Based on these observations, we conclude that the distribution of lipids across the hippocampus and PFC may dictate regional susceptibility to stress and explain the neuromodulatory effects of EA. However, the influence of behavioral testing and different EA parameters on brain lipids, as well as the precise mechanism through which EA regulates lipid composition, require further investigation.

## Data Availability Statement

The original contributions presented in the study are included in the article/Supplementary Material, further inquiries can be directed to the corresponding authors.

## Ethics Statement

The animal study was reviewed and approved by Animal Use and Protection Committee of the Fourth Military Medical University.

## Author Contributions

C-HZ, FX, Q-QS, S-SX, TZ, X-XM, L-SY, and CL were responsible for collecting the data and behavioral evaluation. Z-WP and H-NW financed and designed the study and supervised the data collection and analysis. C-HZ analyzed the data and wrote the first draft with Z-WP and FX. All other authors provided data, reviewed the results, and contributed to the final draft of the report. All authors contributed to the article and approved the submitted version.

## Conflict of Interest

The authors declare that the research was conducted in the absence of any commercial or financial relationships that could be construed as a potential conflict of interest.

## Publisher’s Note

All claims expressed in this article are solely those of the authors and do not necessarily represent those of their affiliated organizations, or those of the publisher, the editors and the reviewers. Any product that may be evaluated in this article, or claim that may be made by its manufacturer, is not guaranteed or endorsed by the publisher.

## Supplementary Material

Supplementary Figure S1 Number of identified lipid subgroups and lipid molecules in this experiment according to the International Lipid Classification and Nomenclature Committee, and based on Lipid Search software version 4.1.

Supplementary Figure S2 **(A)** Scatter plot of PLS-DA model for four groups in hippocamps. **(B)** Coverage of lipids in all three comparisons in hippocamps. **(C)** Number of differential lipids in hippocamps. **(D)** Scatter plot of PLS-DA model for four groups in PFC. **(E)** Coverage of lipids in all three comparisons in PFC. **(F)** Number of differential lipids in PFC.

Supplementary Table S1 Effect of mSPS and EA on behavior in mice.

Supplementary Table S2 Effect of mSPS and EA on the composition of lipid class in the hippocampus and PFC.

Supplementary Table S3 Correlation between PTSD-like behaviors and levels of lipid species in the hippocampus and PFC.

Supplementary Table S4 Characterization of lipids in the hippocampus and prefrontal cortex.

Supplementary Table S5 Normalized lipids after EA treatment in the hippocampus and PFC.

Supplementary Table S6 Correlation between PTSD-like behaviors and levels of lipid species in the hippocampus and PFC.

## Abbreviations

PTSD: post-traumatic stress disorder
EA: electroacupuncture
PFC: prefrontal cortex
mSPS: modified single prolonged stress
HPLC–MS/MS: high performance liquid chromatography–tandem mass spectrometry
OFT: open field test
EPMT: elevated-plus maze test
FCR: fear conditioning response test
MTBE: methyl tert-butyl ether
SD: standard deviation
ANOVA: one-way analysis of variance
DG: diglyceride
TG: triglyceride
SM: sphingomyelin
Cer: ceramides
GM1: gangliosides
CL: cardiolipin
LPG: lysophosphatidylglycerol
LPI: lysophosphatidylinositol
LPS: lysophosphatidylserine
PA: phosphatidic acid
PE: phosphatidylethanolamine
PG: phosphatidylglycerol
PI: phosphatidylinositol
AcCa: acylcarnitine
SQDG: sulfoquinovosyldiacylglycerol
MGDG: monogalactosyldiacylglycerol
Co: coenzyme
So: sphingosine
FA: fatty acid
DGDG: digalactosyldiacylglycerol
LPC: lysophosphatidylcholine
PC: phosphatidylcholine
WE: wax esters
CerG2GNAc1: simple Glc series
phSM: sphingomyelin
PS: phosphatidylserine
CerG1: glucosylceramides
ASM: acid sphingomyelinase
NSM: neutral sphingomyelinase.
