# Supplementary figures and images for: The Impact of Electroacupuncture Early Intervention on the Brain Lipidome in a Mouse Model of Post-traumatic Stress Disorder
Source: Front Mol Neurosci. 2022 Feb 10;15:812479. doi: 10.3389/fnmol.2022.812479 (PMC8866946; doi:10.3389/fnmol.2022.812479)

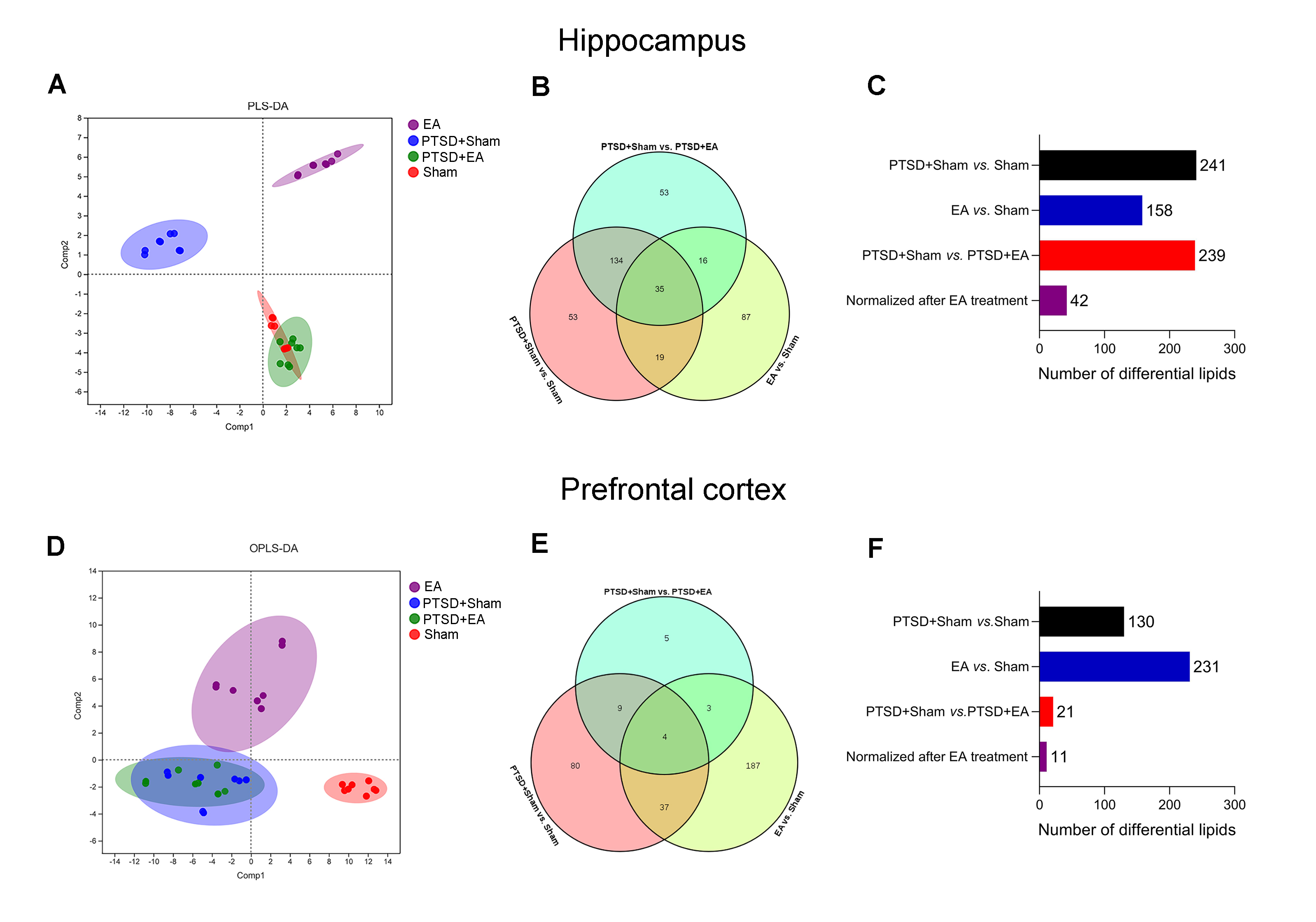

Supplement: Supplementary Figure S2 — (A) Scatter plot of PLS-DA model for four groups in hippocamps. (B) Coverage of lipids in all three comparisons in hippocamps. (C) Number of differential lipids in hippocamps. (D) Scatter plot of PLS-DA model for four groups in PFC. (E) Coverage of lipids in all three comparisons in PFC. (F) Number of differential lipids in PFC. [file Image_2.JPEG]
