# Supplementary material for: The Impact of Electroacupuncture Early Intervention on the Brain Lipidome in a Mouse Model of Post-traumatic Stress Disorder
Source: Front Mol Neurosci. 2022 Feb 10;15:812479. doi: 10.3389/fnmol.2022.812479 (PMC8866946; doi:10.3389/fnmol.2022.812479)
Supplement: Supplementary Table S1 — Effect of mSPS and EA on behavior in mice. [file Table_1.DOCX]

**Table S1. Effect of mSPS and EA on behavior in mice**

| **Behavioral index** | **mSPS factor** | | **EA factor** | |
| --- | --- | --- | --- | --- |
|  | F | *P* | F | *P* |
| Total distance in OFT | 2.320 | 0.139 | 0.270 | 0.871 |
| Time spent in the center (OFT) | 4.348 | 0.046 | 3.659 | 0.066 |
| Time spent in the open arms (OFT) | 10.401 | < 0.01 | 7.373 | 0.011 |
| Contextual freezing time | 10.587 | < 0.01 | 3.365 | 0.077 |
| Cued freezing time | 18.389 | < 0.01 | 1.738 | 0.198 |
