# Supplementary material for: The Impact of Electroacupuncture Early Intervention on the Brain Lipidome in a Mouse Model of Post-traumatic Stress Disorder
Source: Front Mol Neurosci. 2022 Feb 10;15:812479. doi: 10.3389/fnmol.2022.812479 (PMC8866946; doi:10.3389/fnmol.2022.812479)
Supplement: Supplementary Table S2 — Effect of mSPS and EA on the composition of lipid class in the hippocampus and PFC. [file Table_2.DOCX]

**Table S2. Effect of mSPS and EA on the composition of lipid class in the hippocampus and PFC**

| **Lipids** | **mSPS factor** | | **EA factor** | |
| --- | --- | --- | --- | --- |
|  | F | *P* | F | *P* |
| **Hippocampus** |  |  |  |  |
| AcCa | 23.807 | < 0.001 | 18.013 | < 0.001 |
| FA | < 0.001 | .990 | 34.261 | < 0.001 |
| WE | 7.610 | .010 | .960 | .336 |
| DG | 44.152 | < 0.001 | 1.743 | .198 |
| TG | 6.226 | .019 | 60.011 | < 0.001 |
| CL | 28.410 | < 0.001 | 44.882 | < 0.001 |
| LPC | 3.997 | .055 | 0.645 | .429 |
| LPE | 1.662 | .208 | 5.653 | .024 |
| LPG | 13.624 | .001 | 13.004 | .001 |
| LPI | 11.034 | < 0.001 | 0.112 | 0.741 |
| LPS | 14.791 | .001 | 30.514 | < 0.001 |
| PA | 12.734 | .001 | 4.757 | .038 |
| PC | 0.997 | 0.327 | 3.329 | 0.079 |
| PE | 37.813 | < 0.001 | 19.787 | < 0.001 |
| PG | 37.426 | < 0.001 | 19.259 | < 0.001 |
| PI | 16.968 | < 0.001 | 0.004 | 0.952 |
| PS | 0.022 | .884 | 0.027 | .870 |
| Cer | 5.807 | .023 | 14.462 | .001 |
| CerG1 | 1.318 | 0.261 | 0.039 | 0.844 |
| CerG2GNAc1 | 2.735 | 0.109 | 0.221 | .642 |
| GM1 | 9.702 | .004 | 12.357 | .002 |
| phSM | 2.556 | .121 | .641 | .430 |
| SM | 20.162 | < 0.001 | 43.338 | < 0.001 |
| So | 3.842 | .060 | 15.388 | .001 |
| Co | 45.690 | .000 | 22.278 | < 0.001 |
| DGDG | 1.012 | .323 | 27.467 | < 0.001 |
| MGMG | .277 | .603 | 3.843 | .060 |
| SQDG | 21.967 | < 0.001 | 43.754 | < 0.001 |
| MGDG | 47.718 | < 0.001 | 95.459 | < 0.001 |
| **Prefrontal cortex** |  |  |  |  |
| AcCa | 10.957 | .003 | .889 | .354 |
| FA | 23.768 | < 0.001 | 17.552 | < 0.001 |
| WE | 16.173 | < 0.001 | 27.347 | < 0.001 |
| DG | 44.152 | < 0.001 | 1.743 | .198 |
| TG | .125 | .726 | 1.781 | .193 |
| CL | 16.853 | < 0.001 | .841 | .367 |
| LPC | 6.149 | .019 | 20.130 | .000 |
| LPE | 15.163 | < 0.01 | 0.185 | .670 |
| LPG | 1.825 | .188 | 2.289 | .141 |
| LPI | 20.337 | < 0.001 | 47.752 | < 0.001 |
| LPS | 14.209 | .001 | 10.774 | .003 |
| PA | 1.621 | .213 | 16.878 | < 0.001 |
| PC | 31.914 | < 0.001 | 25.379 | < 0.001 |
| PE | 12.559 | .001 | 17.896 | < 0.001 |
| PG | 59.824 | < 0.001 | 38.679 | < 0.001 |
| PI | 23.150 | < 0.001 | 2.782 | .106 |
| PS | 134.871 | < 0.001 | 16.869 | < 0.001 |
| Cer | 16.882 | < 0.001 | 4.422 | .045 |
| CerG1 | 3.731 | .064 | 9.916 | .004 |
| CerG2GNAc1 | 6.514 | .016 | .395 | .535 |
| GM1 | 12.544 | .001 | 14.835 | .001 |
| phSM | 15.960 | < 0.001 | 27.825 | < 0.001 |
| SM | .276 | .604 | 14.343 | .001 |
| So | 6.030 | .021 | 11.571 | .002 |
| Co | 4.069 | .053 | .033 | .857 |
| DGDG | 3.302 | .080 | 3.070 | .091 |
| MGMG | 2.051 | .163 | 33.097 | < 0.001 |
| SQDG | 10.472 | .003 | 2.860 | .102 |
| MGDG | 12.354 | .002 | 11.254 | .002 |
