# Supplementary material for: The Impact of Electroacupuncture Early Intervention on the Brain Lipidome in a Mouse Model of Post-traumatic Stress Disorder
Source: Front Mol Neurosci. 2022 Feb 10;15:812479. doi: 10.3389/fnmol.2022.812479 (PMC8866946; doi:10.3389/fnmol.2022.812479)
Supplement: Supplementary Table S3 — Correlation between PTSD-like behaviors and levels of lipid species in the hippocampus and PFC. [file Table_3.docx]

| **Table S3. Correlation between PTSD-like behaviors and levels of lipid species in the hippocampus and PFC** | | | | | | | | |
| --- | --- | --- | --- | --- | --- | --- | --- | --- |
| Lipid | Time in center (s) | | Time in open arms (s) | | Contextual freezing time(s) | | Cued freezing time(s) | |
|  | r value | *P* value | r value | *P* value | r value | *P* value | r value | *P* value |
| **Hippocampus** |  |  |  |  |  |  |  |  |
| AcCa | 0.399 | 0.024 | 0.327 | 0.068 | -0.557 | 0.001 | -0.416 | 0.018 |
| FA | 0.347 | 0.052 | 0.357 | 0.045 | -0.376 | 0.034 | -0.381 | 0.031 |
| WE | -0.301 | 0.095 | -0.164 | 0.369 | -0.002 | 0.993 | -0.177 | 0.333 |
| DG | -0.255 | 0.159 | -0.504 | 0.003 | 0.479 | 0.006 | 0.528 | 0.002 |
| TG | -0.433 | 0.013 | -0.594 | < 0.001 | 0.329 | 0.066 | 0.331 | 0.064 |
| CL | 0.516 | 0.003 | 0.526 | 0.002 | -0.535 | 0.002 | -0.443 | 0.011 |
| LPC | -0.092 | 0.618 | -0.388 | 0.028 | 0.352 | 0.048 | 0.392 | 0.027 |
| LPE | 0.177 | 0.332 | 0.267 | 0.140 | -0.229 | 0.207 | -0.196 | 0.281 |
| LPG | 0.244 | 0.217 | 0.371 | 0.037 | -0.377 | 0.034 | -0.206 | 0.258 |
| LPI | 0.373 | 0.036 | 0.43 | 0.014 | -0.408 | 0.021 | -0.496 | 0.004 |
| LPS | -0.408 | 0.02 | -0.489 | 0.005 | 0.613 | < 0.001 | 0.527 | 0.002 |
| PA | -0.002 | 0.989 | -0.07 | 0.703 | -0.027 | 0.885 | -0.135 | 0.462 |
| PC | 0.089 | 0.629 | 0.358 | 0.044 | -0.009 | 0.959 | -0.205 | 0.260 |
| PE | -0.379 | 0.032 | -0.61 | < 0.001 | 0.608 | < 0.001 | 0.679 | < 0.001 |
| PG | 0.103 | 0.576 | 0.338 | 0.059 | -0.126 | 0.492 | -0.075 | 0.682 |
| PI | -0.288 | 0.109 | -0.34 | 0.057 | 0.372 | 0.036 | 0.441 | 0.012 |
| PS | 0.082 | 0.655 | -0.167 | 0.361 | 0.121 | 0.508 | 0.229 | 0.207 |
| Cer | -0.155 | 0.396 | -0.581 | < 0.001 | 0.271 | 0.133 | 0.361 | 0.042 |
| CerG1 | -0.23 | 0.900 | 0.162 | 0.377 | -0.088 | 0.632 | -0.08 | 0.661 |
| CerG2GNAc1 | 0.295 | 0.101 | 0.083 | 0.651 | -0.051 | 0.782 | -0.122 | 0.506 |
| GM1 | -0.225 | 0.215 | -0.485 | 0.005 | 0.308 | 0.086 | 0.150 | 0.413 |
| phSM | 0.18 | 0.325 | -0.285 | 0.114 | 0.150 | 0.412 | 0.148 | 0.420 |
| SM | 0.415 | 0.018 | 0.442 | 0.011 | -0.467 | 0.007 | -0.304 | 0.091 |
| So | 0.004 | 0.983 | 0.132 | 0.472 | -0.223 | 0.220 | -0.085 | 0.644 |
| Co | 0.400 | 0.023 | 0.562 | 0.001 | -0.587 | < 0.001 | -0.604 | < 0.001 |
| DGDG | 0.432 | 0.013 | 0.451 | 0.01 | -0.124 | 0.500 | -0.067 | 0.714 |
| MGMG | 0.127 | 0.489 | 0.185 | 0.311 | 0.111 | 0.547 | 0.084 | 0.649 |
| SQDG | 0.119 | 0.517 | 0.293 | 0.103 | -0.065 | 0.726 | -0.055 | 0.767 |
| MDDG | 0.219 | 0.229 | 0.303 | 0.092 | -0.136 | 0.458 | -0.149 | 0.416 |
|  |  |  |  |  |  |  |  |  |
| **Prefrontal cortex** |  |  |  |  |  |  |  |  |
| AcCa | 0.369 | 0.037 | 0.143 | 0.434 | -0.390 | 0.027 | -0.252 | 0.163 |
| FA | 0.353 | 0.048 | 0.509 | 0.003 | -0.123 | 0.504 | -0.209 | 0.251 |
| WE | 0.042 | 0.819 | 0.270 | 0.135 | -0.107 | 0.559 | -0.078 | 0.672 |
| DG | -0.255 | 0.159 | -0.504 | 0.003 | 0.479 | 0.006 | 0.528 | 0.002 |
| TG | -0.015 | 0.935 | 0.078 | 0.672 | -0.198 | 0.276 | -0.077 | 0.675 |
| CL | 0.424 | 0.003 | 0.411 | 0.019 | -0.277 | 0.124 | -0.335 | 0.061 |
| LPC | -0.159 | 0.386 | -0.017 | 0.927 | 0.154 | 0.399 | 0.335 | 0.061 |
| LPE | -0.393 | 0.026 | -0.290 | 0.107 | 0.344 | 0.054 | 0.639 | < 0.001 |
| LPG | 0.089 | 0.629 | 0.171 | 0.351 | -0.556 | 0.001 | -0.390 | 0.027 |
| LPI | 0.167 | 0.362 | 0.256 | 0.157 | -0.271 | 0.133 | -0.178 | 0.329 |
| LPS | 0.269 | 0.137 | 0.034 | 0.853 | -0.401 | 0.023 | -0.403 | 0.022 |
| PA | 0.130 | 0.479 | -0.061 | 0.740 | -0.021 | 0.911 | -0.204 | 0.263 |
| PC | 0.089 | 0.629 | 0.320 | 0.074 | -0.343 | 0.055 | -0.253 | 0.163 |
| PE | 0.281 | 0.120 | 0.340 | 0.057 | -0.201 | 0.271 | -0.190 | 0.299 |
| PG | 0.232 | 0.202 | 0.520 | 0.002 | -0.472 | 0.006 | -0.409 | 0.020 |
| PI | 0.208 | 0.109 | 0.296 | 0.100 | -0.340 | 0.057 | -0.321 | 0.073 |
| PS | 0.364 | 0.041 | 0.340 | 0.057 | -0.362 | 0.042 | -0.478 | 0.006 |
| Cer | 0.299 | 0.396 | 0.332 | 0.064 | -0.508 | 0.003 | -0.368 | 0.038 |
| CerG1 | 0.200 | 0.900 | 0.232 | 0.201 | 0.021 | 0.910 | 0.045 | 0.805 |
| CerG2GNAc1 | -0.043 | 0.101 | -0.223 | 0.219 | 0.134 | 0.463 | 0.242 | 0.181 |
| GM1 | 0.226 | 0.214 | 0.247 | 0.172 | -0.175 | 0.338 | -0.134 | 0.463 |
| phSM | -0.023 | 0.902 | 0.399 | 0.024 | -0.354 | 0.047 | -0.264 | 0.144 |
| SM | -0.131 | 0.474 | 0.119 | 0.515 | -0.116 | 0.526 | -0.017 | 0.927 |
| So | 0.192 | 0.292 | 0.302 | 0.093 | -0.332 | 0.064 | -0.323 | 0.072 |
| Co | 0.273 | 0.130 | 0.373 | 0.036 | -0.355 | 0.046 | -0.253 | 0.162 |
| DGDG | 0.148 | 0.420 | 0.120 | 0.513 | -0.256 | 0.158 | -0.253 | 0.163 |
| MGMG | 0.043 | 0.817 | 0.082 | 0.655 | 0.099 | 0.591 | 0.124 | 0.499 |
| SQDG | 0.148 | 0.419 | 0.309 | 0.085 | -0.362 | 0.042 | -0.295 | 0.101 |
| MGDG | 0.219 | 0.229 | 0.254 | 0.161 | -0.167 | 0.360 | -0.194 | 0.287 |
