# Supplementary material for: The Impact of Electroacupuncture Early Intervention on the Brain Lipidome in a Mouse Model of Post-traumatic Stress Disorder
Source: Front Mol Neurosci. 2022 Feb 10;15:812479. doi: 10.3389/fnmol.2022.812479 (PMC8866946; doi:10.3389/fnmol.2022.812479)
Supplement: Supplementary Table S4 — Characterization of lipids in the hippocampus and prefrontal cortex. [file Table_4.docx]

|  | **Table S4. Characterization of lipids in the hippocampus and** **prefrontal cortex** | | | | |
| --- | --- | --- | --- | --- | --- |
| **Brain region** | **Comparison between groups** | **LipidIon** | **Class** | **Fold change** | ***P* value** |
| **Hippocampus** | **PTSD+Sham *vs.* Sham** | PC(33:0)+H | PC | 0.133 | < 0.001 |
|  |  | DG(34:1e)+Na | DG | 0.154 | < 0.001 |
|  |  | CL(18:2/20:4/16:0/20:4)-H | CL | 0.263 | < 0.001 |
|  |  | LPI(18:0)-H | LPI | 0.419 | < 0.001 |
|  |  | PC(34:1)+H | PC | 0.421 | < 0.001 |
|  |  | CL(22:6/18:1/16:0/20:4)-H | CL | 0.422 | < 0.001 |
|  |  | PS(18:3/20:4)-H | PS | 0.427 | < 0.001 |
|  |  | PC(56:5)+H | PC | 0.469 | < 0.001 |
|  |  | PG(39:6)-H | PG | 0.486 | < 0.001 |
|  |  | PC(26:2p)+H | PC | 0.511 | < 0.001 |
|  |  | SM(d22:1/16:0)+HCOO | SM | 0.511 | < 0.001 |
|  |  | MGDG(10:4/22:6)+HCOO | MGDG | 0.516 | < 0.001 |
|  |  | AcCa(20:4)+H | AcCa | 0.526 | < 0.001 |
|  |  | PS(16:0/20:4)-H | PS | 0.537 | < 0.001 |
|  |  | AcCa(22:0)+H | AcCa | 0.537 | < 0.001 |
|  |  | SM(d34:1)+H | SM | 0.538 | < 0.001 |
|  |  | PS(18:1/18:1)-H | PS | 0.541 | < 0.001 |
|  |  | PC(60:5)+H | PC | 0.553 | < 0.001 |
|  |  | Co(Q9)+NH4 | Co | 0.556 | < 0.001 |
|  |  | PC(38:2e)+H | PC | 0.560 | < 0.001 |
|  |  | CL(22:6/22:6/22:6/20:4)-H | CL | 0.560 | < 0.001 |
|  |  | PC(18:0/20:1)+HCOO | PC | 0.566 | < 0.001 |
|  |  | CL(22:6/22:6/16:1/20:4)-H | CL | 0.570 | < 0.001 |
|  |  | FA(22:6)-H | FA | 0.571 | < 0.001 |
|  |  | PC(20:0p/16:0)+H | PC | 0.572 | < 0.001 |
|  |  | CL(18:4/22:6/20:4/18:1)-H | CL | 0.578 | < 0.001 |
|  |  | SM(d36:0)+H | SM | 0.578 | 0.001 |
|  |  | PC(58:5)+H | PC | 0.580 | < 0.001 |
|  |  | CL(18:1/16:0/16:0/18:1)-H | CL | 0.582 | 0.001 |
|  |  | PC(58:6)+H | PC | 0.582 | < 0.001 |
|  |  | PS(18:0/18:1)-H | PS | 0.589 | < 0.001 |
|  |  | MGDG(18:3/18:3)+HCOO | MGDG | 0.590 | < 0.001 |
|  |  | LPS(18:1)-H | LPS | 0.593 | < 0.001 |
|  |  | CL(18:2/18:1/16:1/18:1)-H | CL | 0.595 | < 0.001 |
|  |  | CL(22:6/20:4/22:6/22:6)-H | CL | 0.597 | < 0.001 |
|  |  | LPE(18:0)-H | LPE | 0.598 | < 0.001 |
|  |  | PS(18:1/18:1)+H | PS | 0.602 | < 0.001 |
|  |  | PC(44:4)+H | PC | 0.605 | < 0.001 |
|  |  | CL(20:4/16:0/16:1/20:4)-H | CL | 0.608 | < 0.001 |
|  |  | PC(34:2e)+H | PC | 0.610 | < 0.001 |
|  |  | PS(16:0/18:1)-H | PS | 0.613 | < 0.001 |
|  |  | CerG1(d18:1/24:1)+H | CerG1 | 0.619 | 0.007 |
|  |  | CL(18:1/16:1/16:1/18:1)-H | CL | 0.622 | < 0.001 |
|  |  | PC(42:8)+H | PC | 0.622 | < 0.001 |
|  |  | PC(39:1)+H | PC | 0.627 | < 0.001 |
|  |  | AcCa(22:6)+H | AcCa | 0.630 | < 0.001 |
|  |  | CL(18:2/20:4/16:1/20:4)-H | CL | 0.633 | < 0.001 |
|  |  | PC(34:5)+H | PC | 0.640 | < 0.001 |
|  |  | TG(16:0/18:1/18:2)+NH4 | TG | 0.641 | < 0.001 |
|  |  | PC(39:0)+H | PC | 0.643 | 0.001 |
|  |  | CL(18:1/18:1/18:1/20:4)-H | CL | 0.644 | < 0.001 |
|  |  | CerG1(d18:1/22:0+O)+H | CerG1 | 0.647 | 0.004 |
|  |  | CL(18:2/18:1/18:1/18:2)-H | CL | 0.647 | < 0.001 |
|  |  | PC(44:8)+H | PC | 0.650 | < 0.001 |
|  |  | PC(43:4)+H | PC | 0.651 | < 0.001 |
|  |  | PC(35:4)+H | PC | 0.654 | < 0.001 |
|  |  | PS(18:0/22:4)-H | PS | 0.658 | 0.001 |
|  |  | PS(16:0/22:6)-H | PS | 0.665 | < 0.001 |
|  |  | TG(16:0/12:0/18:1)+NH4 | TG | 0.667 | < 0.001 |
|  |  | PC(54:5)+H | PC | 0.668 | < 0.001 |
|  |  | TG(15:0/16:0/16:0)+NH4 | TG | 1.506 | < 0.001 |
|  |  | LPE(20:1)-H | LPE | 1.508 | < 0.001 |
|  |  | CerG1(d18:1/25:2)+H | CerG1 | 1.509 | < 0.001 |
|  |  | CerG1(d18:1/18:1)+H | CerG1 | 1.512 | < 0.001 |
|  |  | PE(36:2p)-H | PE | 1.515 | < 0.001 |
|  |  | PC(32:1/20:4)+HCOO | PC | 1.515 | < 0.001 |
|  |  | PE(18:1/18:1)-H | PE | 1.517 | < 0.001 |
|  |  | CerG1(d58:4)+H | CerG1 | 1.518 | < 0.001 |
|  |  | TG(16:0/18:1/24:0)+NH4 | TG | 1.519 | < 0.001 |
|  |  | PS(36:3p)-H | PS | 1.521 | < 0.001 |
|  |  | PE(20:0p/16:0)-H | PE | 1.522 | < 0.001 |
|  |  | PE(20:1/22:4)-H | PE | 1.523 | < 0.001 |
|  |  | PS(44:11)-H | PS | 1.524 | < 0.001 |
|  |  | Cer(d18:0+pO/24:0+O)+HCOO | Cer | 1.526 | 0.008 |
|  |  | PE(18:0/20:2)-H | PE | 1.531 | < 0.001 |
|  |  | Cer(d36:3+O)+H | Cer | 1.533 | 0.042 |
|  |  | PE(18:0/20:4)+H | PE | 1.534 | < 0.001 |
|  |  | PE(54:2)-H | PE | 1.535 | < 0.001 |
|  |  | PE(18:0/20:1)-H | PE | 1.538 | < 0.001 |
|  |  | PS(40:6p)-H | PS | 1.544 | < 0.001 |
|  |  | Cer(d18:1/24:2)+H | Cer | 1.547 | < 0.001 |
|  |  | Cer(d18:1/24:3)+H | Cer | 1.547 | 0.001 |
|  |  | Cer(d18:2/24:0+O)+H | Cer | 1.549 | < 0.001 |
|  |  | CerG1(d18:1/25:0)+H | CerG1 | 1.553 | < 0.001 |
|  |  | PE(18:0/18:1)-H | PE | 1.554 | < 0.001 |
|  |  | DG(16:0/22:4)+NH4 | DG | 1.557 | < 0.001 |
|  |  | LPC(24:1)+HCOO | LPC | 1.560 | < 0.001 |
|  |  | PE(36:1/22:6)-H | PE | 1.568 | < 0.001 |
|  |  | PE(16:1p/22:6)-H | PE | 1.571 | < 0.001 |
|  |  | Cer(d17:1/27:2)+HCOO | Cer | 1.575 | < 0.001 |
|  |  | PE(17:0/18:1)-H | PE | 1.577 | < 0.001 |
|  |  | MGMG(18:1)+HCOO | MGMG | 1.581 | 0.001 |
|  |  | PS(20:4/22:6)-H | PS | 1.583 | < 0.001 |
|  |  | PI(16:0/22:4)-H | PI | 1.585 | < 0.001 |
|  |  | Cer(d18:1/25:2)+H | Cer | 1.591 | < 0.001 |
|  |  | CerG1(d18:0/24:0+O)+H | CerG1 | 1.591 | 0.001 |
|  |  | Cer(d18:1/21:1)+H | Cer | 1.593 | < 0.001 |
|  |  | PI(18:0/18:1)-H | PI | 1.595 | < 0.001 |
|  |  | LPS(22:6)-H | LPS | 1.598 | < 0.001 |
|  |  | PE(35:1p)-H | PE | 1.598 | < 0.001 |
|  |  | Cer(d18:1/23:1)+H | Cer | 1.599 | < 0.001 |
|  |  | SM(d41:0)+H | SM | 1.604 | 0.010 |
|  |  | PE(37:2p)-H | PE | 1.605 | < 0.001 |
|  |  | Cer(d18:1/24:1)+H | Cer | 1.607 | 0.003 |
|  |  | CerG1(d18:1/24:1)+H | CerG1 | 1.610 | 0.001 |
|  |  | PE(18:0p/20:0)-H | PE | 1.615 | < 0.001 |
|  |  | PS(38:3p)-H | PS | 1.617 | < 0.001 |
|  |  | CerG1(d18:1/21:1)+H | CerG1 | 1.618 | < 0.001 |
|  |  | PE(19:1)+H | PE | 1.620 | < 0.001 |
|  |  | CerG1(d18:1/26:2)+H | CerG1 | 1.621 | < 0.001 |
|  |  | CerG1(d18:0/22:0+O)+H | CerG1 | 1.623 | 0.002 |
|  |  | LPC(20:0)+HCOO | LPC | 1.624 | < 0.001 |
|  |  | TG(18:0/16:0/18:0)+NH4 | TG | 1.624 | 0.018 |
|  |  | Cer(d18:1/23:0)+H | Cer | 1.626 | < 0.001 |
|  |  | Cer(d18:1/24:0+O)+H | Cer | 1.636 | < 0.001 |
|  |  | DG(18:0/22:4)+NH4 | DG | 1.643 | < 0.001 |
|  |  | CerG1(d18:1/24:0+O)+H | CerG1 | 1.651 | < 0.001 |
|  |  | PE(18:1/22:6)-H | PE | 1.652 | < 0.001 |
|  |  | CerG1(d18:1/24:1)+H | CerG1 | 1.653 | 0.001 |
|  |  | CerG1(d18:0+pO/25:2)+H | CerG1 | 1.654 | < 0.001 |
|  |  | Cer(d20:0/18:0)+H | Cer | 1.657 | < 0.001 |
|  |  | CerG1(d18:2/23:1)+H | CerG1 | 1.661 | < 0.001 |
|  |  | Cer(d18:1/22:0+O)+H | Cer | 1.664 | < 0.001 |
|  |  | PE(23:4)+H | PE | 1.667 | < 0.001 |
|  |  | CerG1(d18:1/24:0)+H | CerG1 | 1.668 | 0.017 |
|  |  | CerG1(d18:1/21:0+O)+H | CerG1 | 1.669 | < 0.001 |
|  |  | PS(18:0/22:6)-H | PS | 1.671 | < 0.001 |
|  |  | PE(18:1p/21:1)-H | PE | 1.674 | < 0.001 |
|  |  | CerG1(d18:1/18:0+O)+H | CerG1 | 1.675 | < 0.001 |
|  |  | CerG1(d18:0/22:0+O)+H | CerG1 | 1.680 | < 0.001 |
|  |  | PS(20:2/22:6)-H | PS | 1.680 | < 0.001 |
|  |  | PE(18:0p/23:1)+H | PE | 1.684 | < 0.001 |
|  |  | CerG1(d18:1/26:1)+H | CerG1 | 1.686 | < 0.001 |
|  |  | TG(18:1/18:1/18:1)+NH4 | TG | 1.697 | < 0.001 |
|  |  | PE(16:0p/18:1)+H | PE | 1.707 | < 0.001 |
|  |  | CerG1(d18:1/24:0)+H | CerG1 | 1.708 | < 0.001 |
|  |  | CerG1(d18:1/20:1)+H | CerG1 | 1.710 | < 0.001 |
|  |  | PC(40:3)+H | PC | 1.713 | < 0.001 |
|  |  | Cer(d18:1/24:1)+H | Cer | 1.714 | 0.001 |
|  |  | DG(18:1/24:1)+NH4 | DG | 1.715 | < 0.001 |
|  |  | CerG1(d18:1/26:0+O)+H | CerG1 | 1.720 | < 0.001 |
|  |  | LPC(22:0)+HCOO | LPC | 1.723 | < 0.001 |
|  |  | CerG1(d18:2/24:0+O)+H | CerG1 | 1.729 | < 0.001 |
|  |  | CerG1(d18:0/25:0+O)+H | CerG1 | 1.731 | < 0.001 |
|  |  | Cer(d13:0/17:1)+HCOO | Cer | 1.731 | < 0.001 |
|  |  | CerG1(d40:0+pO+O)+H | CerG1 | 1.732 | 0.001 |
|  |  | MGDG(18:1/20:1)+HCOO | MGDG | 1.737 | < 0.001 |
|  |  | CerG1(d18:0+pO/26:2)+H | CerG1 | 1.743 | < 0.001 |
|  |  | Cer(d18:1+hO/24:0)+HCOO | Cer | 1.746 | < 0.001 |
|  |  | SM(d43:1)+H | SM | 1.748 | 0.017 |
|  |  | Cer(d30:0)+H | Cer | 1.756 | < 0.001 |
|  |  | CerG1(d18:1/23:0)+H | CerG1 | 1.760 | < 0.001 |
|  |  | PE(16:1p/22:6)+H | PE | 1.764 | < 0.001 |
|  |  | SM(d38:4)+H | SM | 1.772 | < 0.001 |
|  |  | Cer(d18:1/24:0)+H | Cer | 1.773 | < 0.001 |
|  |  | Cer(d18:1/24:1)+H | Cer | 1.774 | < 0.001 |
|  |  | Cer(d18:1+hO/23:0)+HCOO | Cer | 1.774 | < 0.001 |
|  |  | PE(44:1)-H | PE | 1.777 | < 0.001 |
|  |  | Cer(d18:0/23:0+O)+H | Cer | 1.791 | < 0.001 |
|  |  | Cer(d30:3)+H | Cer | 1.793 | 0.002 |
|  |  | PS(22:5/22:6)-H | PS | 1.794 | < 0.001 |
|  |  | CerG1(d18:1/20:0+O)+H | CerG1 | 1.795 | < 0.001 |
|  |  | DG(20:4/20:4)+NH4 | DG | 1.808 | 0.001 |
|  |  | PE(42:2)-H | PE | 1.811 | < 0.001 |
|  |  | Cer(d18:1/24:1)+H | Cer | 1.812 | < 0.001 |
|  |  | CerG1(d18:1/24:1)+H | CerG1 | 1.818 | < 0.001 |
|  |  | CerG1(d18:1/26:0)+H | CerG1 | 1.824 | < 0.001 |
|  |  | PE(18:1p/22:5)-H | PE | 1.827 | < 0.001 |
|  |  | Cer(d20:1)+H | Cer | 1.830 | 0.006 |
|  |  | CerG1(d18:1/20:0)+H | CerG1 | 1.832 | < 0.001 |
|  |  | PE(38:2p)-H | PE | 1.836 | < 0.001 |
|  |  | DG(18:0/20:3)+NH4 | DG | 1.838 | < 0.001 |
|  |  | PG(38:1p)-H | PG | 1.840 | < 0.001 |
|  |  | CerG1(d18:1/22:1)+H | CerG1 | 1.850 | < 0.001 |
|  |  | Cer(d18:0/24:0+O)+H | Cer | 1.854 | < 0.001 |
|  |  | TG(18:1/18:1/18:2)+NH4 | TG | 1.869 | < 0.001 |
|  |  | Cer(d18:1+hO/22:0)+HCOO | Cer | 1.877 | < 0.001 |
|  |  | Cer(d18:0/20:0+O)+H | Cer | 1.877 | < 0.001 |
|  |  | Cer(d18:1/24:0+O)+H | Cer | 1.893 | < 0.001 |
|  |  | CerG1(d18:0/24:0)+H | CerG1 | 1.903 | < 0.001 |
|  |  | PE(18:0p/22:3)+H | PE | 1.909 | < 0.001 |
|  |  | CerG1(d18:0/23:0)+H | CerG1 | 1.916 | < 0.001 |
|  |  | CerG1(d18:1/25:1)+H | CerG1 | 1.917 | < 0.001 |
|  |  | Cer(d18:0/24:0)+H | Cer | 1.940 | < 0.001 |
|  |  | PS(20:3/22:4)-H | PS | 1.942 | < 0.001 |
|  |  | SM(d44:2)+H | SM | 1.955 | < 0.001 |
|  |  | CerG1(d18:1/44:0EO)+H | CerG1 | 1.964 | < 0.001 |
|  |  | TG(16:0/18:1/22:0)+NH4 | TG | 1.978 | < 0.001 |
|  |  | PS(20:3/22:6)-H | PS | 1.990 | < 0.001 |
|  |  | CerG1(d18:1/22:0)+H | CerG1 | 1.991 | < 0.001 |
|  |  | Cer(d18:0/22:0+O)+H | Cer | 2.003 | < 0.001 |
|  |  | CerG1(d18:1/24:2)+H | CerG1 | 2.007 | < 0.001 |
|  |  | Cer(d18:1/22:0)+H | Cer | 2.008 | < 0.001 |
|  |  | TG(16:0/16:0/18:2)+NH4 | TG | 2.014 | < 0.001 |
|  |  | DG(16:0/18:1)+NH4 | DG | 2.031 | < 0.001 |
|  |  | PS(16:1/18:1)-H | PS | 2.031 | < 0.001 |
|  |  | CerG1(d18:0+pO/24:2)+H | CerG1 | 2.041 | < 0.001 |
|  |  | LPC(20:1)+H | LPC | 2.044 | < 0.001 |
|  |  | SM(d38:5)+H | SM | 2.044 | < 0.001 |
|  |  | CerG1(d18:0+pO/24:0)+H | CerG1 | 2.051 | < 0.001 |
|  |  | PS(41:2)-H | PS | 2.062 | < 0.001 |
|  |  | Cer(d34:3)+H | Cer | 2.063 | < 0.001 |
|  |  | PS(38:4p)-H | PS | 2.065 | < 0.001 |
|  |  | Cer(d22:1)+H | Cer | 2.069 | < 0.001 |
|  |  | Cer(d18:0/24:1)+H | Cer | 2.069 | < 0.001 |
|  |  | PS(22:6/22:6)-H | PS | 2.072 | < 0.001 |
|  |  | TG(18:3/18:2/18:2)+NH4 | TG | 2.076 | < 0.001 |
|  |  | CerG1(d18:1/22:0)+H | CerG1 | 2.084 | < 0.001 |
|  |  | TG(19:0/18:1/18:1)+NH4 | TG | 2.086 | < 0.001 |
|  |  | PE(18:1p/20:1)+H | PE | 2.119 | < 0.001 |
|  |  | Cer(d18:1/24:1)+H | Cer | 2.132 | < 0.001 |
|  |  | CerG1(d18:1/24:0+O)+H | CerG1 | 2.145 | < 0.001 |
|  |  | PS(20:2/22:4)-H | PS | 2.153 | < 0.001 |
|  |  | Cer(d18:0/22:0)+H | Cer | 2.175 | < 0.001 |
|  |  | Cer(d18:1+hO/24:1)+HCOO | Cer | 2.183 | < 0.001 |
|  |  | DG(18:1/18:1)+NH4 | DG | 2.207 | < 0.001 |
|  |  | DG(18:0/20:1)+NH4 | DG | 2.219 | < 0.001 |
|  |  | TG(18:1/18:2/22:0)+NH4 | TG | 2.225 | 0.005 |
|  |  | TG(18:1/18:2/24:0)+NH4 | TG | 2.230 | < 0.001 |
|  |  | DG(16:0/18:2)+NH4 | DG | 2.241 | < 0.001 |
|  |  | CerG1(d18:0/22:0)+H | CerG1 | 2.250 | < 0.001 |
|  |  | DG(18:1/22:0)+NH4 | DG | 2.270 | < 0.001 |
|  |  | PE(38:2p)-H | PE | 2.284 | < 0.001 |
|  |  | TG(20:0/18:1/18:1)+NH4 | TG | 2.299 | < 0.001 |
|  |  | DG(18:1/22:1)+NH4 | DG | 2.344 | < 0.001 |
|  |  | PS(18:1/22:6)-H | PS | 2.357 | < 0.001 |
|  |  | TG(16:0/18:1/18:1)+NH4 | TG | 2.376 | < 0.001 |
|  |  | TG(18:1/18:1/21:0)+NH4 | TG | 2.401 | < 0.001 |
|  |  | PE(18:1p/22:6)-H | PE | 2.405 | < 0.001 |
|  |  | LPE(20:2)-H | LPE | 2.512 | < 0.001 |
|  |  | Cer(d18:2/16:0)+HCOO | Cer | 2.550 | < 0.001 |
|  |  | TG(18:1/18:1/24:0)+NH4 | TG | 2.611 | < 0.001 |
|  |  | TG(18:1/18:1/22:0)+NH4 | TG | 2.736 | < 0.001 |
|  |  | PI(18:1/18:1)-H | PI | 2.881 | < 0.001 |
|  |  | CerG1(d18:0/24:1)+H | CerG1 | 2.972 | < 0.001 |
|  |  | TG(18:1/18:1/23:0)+NH4 | TG | 3.196 | < 0.001 |
|  |  | LPC(24:0)+H | LPC | 3.369 | < 0.001 |
|  |  | PS(42:7p)-H | PS | 3.767 | < 0.001 |
|  |  | TG(17:0/18:1/18:1)+NH4 | TG | 3.792 | < 0.001 |
|  |  | TG(56:6)+NH4 | TG | 4.254 | 0.002 |
|  |  | TG(16:0/18:1/20:1)+NH4 | TG | 4.950 | < 0.001 |
|  | **PTSD+Sham *vs.* PTSD+EA** | PC(18:0p/20:1)+HCOO | PC | 0.223 | < 0.001 |
|  |  | FA(22:6)-H | FA | 0.350 | < 0.001 |
|  |  | MGDG(10:4/22:6)+HCOO | MGDG | 0.355 | < 0.001 |
|  |  | CL(22:6/18:1/16:0/20:4)-H | CL | 0.364 | < 0.001 |
|  |  | CL(18:2/20:4/16:0/20:4)-H | CL | 0.437 | < 0.001 |
|  |  | PC(38:1)+H | PC | 0.472 | < 0.001 |
|  |  | PC(16:0/24:0)+HCOO | PC | 0.494 | < 0.001 |
|  |  | PS(18:1/24:0)-H | PS | 0.513 | < 0.001 |
|  |  | CL(22:6/22:6/16:1/20:4)-H | CL | 0.515 | < 0.001 |
|  |  | CL(22:6/20:4/22:6/22:6)-H | CL | 0.529 | < 0.001 |
|  |  | PC(38:2e)+H | PC | 0.535 | < 0.001 |
|  |  | PC(58:6)+H | PC | 0.536 | < 0.001 |
|  |  | SM(d34:1)+H | SM | 0.543 | < 0.001 |
|  |  | MGDG(18:3/18:3)+HCOO | MGDG | 0.544 | 0.001 |
|  |  | PG(39:6)-H | PG | 0.556 | < 0.001 |
|  |  | SM(d42:2)+HCOO | SM | 0.558 | < 0.001 |
|  |  | CL(18:1/16:0/16:0/20:0)-H | CL | 0.562 | < 0.001 |
|  |  | PC(34:1)+H | PC | 0.563 | 0.001 |
|  |  | TG(18:1/18:1/20:4)+NH4 | TG | 0.564 | < 0.001 |
|  |  | PC(26:2p)+H | PC | 0.571 | < 0.001 |
|  |  | CerG1(d18:1/24:1)+H | CerG1 | 0.575 | 0.008 |
|  |  | SM(d22:1/16:0)+HCOO | SM | 0.576 | < 0.001 |
|  |  | LPE(18:0)-H | LPE | 0.577 | < 0.001 |
|  |  | PC(30:1)+H | PC | 0.589 | < 0.001 |
|  |  | Co(Q9)+NH4 | Co | 0.590 | < 0.001 |
|  |  | CL(18:2/18:1/16:1/18:1)-H | CL | 0.591 | < 0.001 |
|  |  | PC(16:0/20:5)+HCOO | PC | 0.592 | < 0.001 |
|  |  | PC(36:2)+H | PC | 0.604 | 0.010 |
|  |  | CL(18:1/18:1/18:1/20:4)-H | CL | 0.610 | < 0.001 |
|  |  | CL(18:2/20:4/16:1/20:4)-H | CL | 0.615 | < 0.001 |
|  |  | PC(42:8)+H | PC | 0.621 | < 0.001 |
|  |  | CL(18:4/22:6/20:4/18:1)-H | CL | 0.623 | < 0.001 |
|  |  | PC(32:0)+H | PC | 0.624 | < 0.001 |
|  |  | CL(18:1/16:0/16:0/18:1)-H | CL | 0.635 | 0.003 |
|  |  | LPC(20:4)+H | LPC | 0.645 | < 0.001 |
|  |  | CL(18:1/16:1/18:1/18:1)-H | CL | 0.648 | < 0.001 |
|  |  | CL(18:2/18:1/18:1/18:2)-H | CL | 0.652 | < 0.001 |
|  |  | PC(32:0e)+H | PC | 0.654 | 0.016 |
|  |  | CL(22:6/22:6/22:6/20:4)-H | CL | 0.655 | 0.009 |
|  |  | CL(18:1/18:1/18:1/16:0)-H | CL | 0.655 | 0.001 |
|  |  | SM(d36:0)+H | SM | 0.657 | 0.005 |
|  |  | PS(16:0/18:1)-H | PS | 0.657 | 0.001 |
|  |  | PC(39:0)+H | PC | 0.659 | < 0.001 |
|  |  | PC(33:1p)+H | PC | 0.660 | 0.016 |
|  |  | CL(20:4/16:0/18:1/20:4)-H | CL | 0.662 | < 0.001 |
|  |  | TG(16:0/22:6/22:6)+NH4 | TG | 0.664 | < 0.001 |
|  |  | LPS(18:1)-H | LPS | 0.666 | < 0.001 |
|  |  | DG(18:1/22:1)+NH4 | DG | 1.501 | 0.005 |
|  |  | TG(16:0/18:1/24:0)+NH4 | TG | 1.501 | < 0.001 |
|  |  | PE(18:2p/20:4)-H | PE | 1.503 | < 0.001 |
|  |  | PE(56:2)-H | PE | 1.504 | < 0.001 |
|  |  | DG(18:1/18:1)+NH4 | DG | 1.506 | 0.001 |
|  |  | Cer(d18:1/25:0+O)+H | Cer | 1.507 | < 0.001 |
|  |  | Cer(d18:2/20:2)+H | Cer | 1.509 | 0.002 |
|  |  | MGDG(18:1/20:1)+HCOO | MGDG | 1.511 | < 0.001 |
|  |  | CerG1(d18:1/18:0)+H | CerG1 | 1.512 | < 0.001 |
|  |  | Cer(d26:0+pO)+H | Cer | 1.523 | 0.020 |
|  |  | PI(16:1/20:4)-H | PI | 1.524 | < 0.001 |
|  |  | PE(35:1p)-H | PE | 1.527 | < 0.001 |
|  |  | PG(42:6)-H | PG | 1.529 | < 0.001 |
|  |  | PE(18:0/18:1)-H | PE | 1.534 | < 0.001 |
|  |  | PE(18:1p/22:5)-H | PE | 1.538 | < 0.001 |
|  |  | PE(16:0e/20:4)-H | PE | 1.546 | < 0.001 |
|  |  | PE(36:1/20:4)-H | PE | 1.549 | < 0.001 |
|  |  | CerG1(d18:2/24:1)+H | CerG1 | 1.557 | < 0.001 |
|  |  | PE(44:1)-H | PE | 1.557 | < 0.001 |
|  |  | PS(16:0/22:6)-H | PS | 1.559 | < 0.001 |
|  |  | Cer(d28:0)+H | Cer | 1.560 | 0.003 |
|  |  | PG(43:2)-H | PG | 1.561 | < 0.001 |
|  |  | CerG1(d18:0+pO/25:2)+H | CerG1 | 1.564 | 0.001 |
|  |  | PS(36:3p)-H | PS | 1.565 | < 0.001 |
|  |  | TG(16:0/17:1/18:1)+NH4 | TG | 1.572 | 0.002 |
|  |  | PE(38:5)+H | PE | 1.575 | < 0.001 |
|  |  | PC(37:0)+H | PC | 1.576 | 0.033 |
|  |  | Cer(d18:0+pO/24:0+O)+HCOO | Cer | 1.576 | 0.004 |
|  |  | Cer(d18:1/25:1)+H | Cer | 1.584 | < 0.001 |
|  |  | Cer(d18:1/22:2)+HCOO | Cer | 1.586 | < 0.001 |
|  |  | PA(35:1)-H | PA | 1.587 | < 0.001 |
|  |  | CerG1(d18:1/26:2)+H | CerG1 | 1.588 | < 0.001 |
|  |  | Cer(d18:1/24:0+O)+H | Cer | 1.593 | < 0.001 |
|  |  | PE(42:2)-H | PE | 1.593 | < 0.001 |
|  |  | PC(42:1e)+H | PC | 1.595 | < 0.001 |
|  |  | PE(40:7)+H | PE | 1.598 | < 0.001 |
|  |  | PE(18:0/18:1)-H | PE | 1.599 | < 0.001 |
|  |  | TG(6:0/14:0/16:0)+NH4 | TG | 1.601 | < 0.001 |
|  |  | Cer(d17:1/27:2)+HCOO | Cer | 1.605 | < 0.001 |
|  |  | LPS(22:6)-H | LPS | 1.606 | < 0.001 |
|  |  | DG(18:0/20:1)+NH4 | DG | 1.609 | < 0.001 |
|  |  | TG(18:1/18:2/22:0)+NH4 | TG | 1.609 | 0.040 |
|  |  | CerG1(d18:0+pO/26:2)+H | CerG1 | 1.610 | < 0.001 |
|  |  | CerG1(d18:1/18:1)+H | CerG1 | 1.611 | < 0.001 |
|  |  | Cer(d18:1/24:1)+H | Cer | 1.624 | 0.005 |
|  |  | CerG1(d18:1/25:1)+H | CerG1 | 1.625 | < 0.001 |
|  |  | CerG1(d18:1/25:0+O)+H | CerG1 | 1.626 | < 0.001 |
|  |  | Cer(d18:1/24:1)+HCOO | Cer | 1.628 | < 0.001 |
|  |  | PE(34:1/20:4)-H | PE | 1.628 | < 0.001 |
|  |  | PC(40:3)+H | PC | 1.629 | < 0.001 |
|  |  | PE(36:0p)-H | PE | 1.630 | < 0.001 |
|  |  | CerG1(d18:1/22:1)+H | CerG1 | 1.638 | 0.002 |
|  |  | PE(16:1p/22:6)-H | PE | 1.638 | < 0.001 |
|  |  | Cer(d18:1+hO/24:1)+HCOO | Cer | 1.646 | 0.005 |
|  |  | CerG1(d58:4)+H | CerG1 | 1.652 | < 0.001 |
|  |  | SM(d41:2)+H | SM | 1.653 | 0.048 |
|  |  | SM(d41:0)+H | SM | 1.655 | 0.006 |
|  |  | PE(38:2p)-H | PE | 1.660 | < 0.001 |
|  |  | PE(18:0/22:5)-H | PE | 1.667 | < 0.001 |
|  |  | PE(18:0p/20:0)-H | PE | 1.670 | < 0.001 |
|  |  | CerG1(d18:1/21:1)+H | CerG1 | 1.677 | < 0.001 |
|  |  | PE(38:2p)-H | PE | 1.680 | < 0.001 |
|  |  | DG(16:0/18:1)+NH4 | DG | 1.684 | 0.002 |
|  |  | Cer(d18:1/24:1)+H | Cer | 1.686 | < 0.001 |
|  |  | CerG1(d18:1/25:1)+H | CerG1 | 1.690 | 0.001 |
|  |  | Cer(d18:1/21:1)+H | Cer | 1.699 | < 0.001 |
|  |  | PE(18:0p/23:1)+H | PE | 1.706 | < 0.001 |
|  |  | CerG1(d18:1/21:0+O)+H | CerG1 | 1.708 | < 0.001 |
|  |  | PS(36:1p)-H | PS | 1.709 | < 0.001 |
|  |  | Cer(d13:0/17:1)+HCOO | Cer | 1.710 | < 0.001 |
|  |  | CerG1(d18:1/24:1)+H | CerG1 | 1.723 | < 0.001 |
|  |  | PE(18:0p/24:1)-H | PE | 1.724 | < 0.001 |
|  |  | PS(42:5)-H | PS | 1.724 | < 0.001 |
|  |  | Cer(d18:1/23:0)+H | Cer | 1.728 | < 0.001 |
|  |  | LPC(22:0)+HCOO | LPC | 1.729 | < 0.001 |
|  |  | PE(16:1p/22:6)+H | PE | 1.733 | < 0.001 |
|  |  | CerG1(d18:1/25:0)+H | CerG1 | 1.749 | < 0.001 |
|  |  | Cer(d18:1/23:0+O)+H | Cer | 1.755 | < 0.001 |
|  |  | CerG1(d18:1/20:1)+H | CerG1 | 1.759 | < 0.001 |
|  |  | TG(16:0/18:1/22:0)+NH4 | TG | 1.761 | < 0.001 |
|  |  | Cer(d20:1)+H | Cer | 1.770 | 0.003 |
|  |  | CerG1(d18:1/20:0+O)+H | CerG1 | 1.787 | < 0.001 |
|  |  | PE(16:0p/18:1)+H | PE | 1.791 | < 0.001 |
|  |  | SM(d43:1)+H | SM | 1.795 | 0.010 |
|  |  | CerG1(d18:1/24:1)+H | CerG1 | 1.802 | < 0.001 |
|  |  | PE(23:4)+H | PE | 1.802 | < 0.001 |
|  |  | CerG1(d18:1/18:0+O)+H | CerG1 | 1.803 | < 0.001 |
|  |  | PE(35:2p)-H | PE | 1.807 | < 0.001 |
|  |  | Cer(d18:1/22:0+O)+H | Cer | 1.815 | < 0.001 |
|  |  | Cer(d18:1/24:0+O)+H | Cer | 1.820 | < 0.001 |
|  |  | CerG1(d40:0+pO+O)+H | CerG1 | 1.827 | 0.001 |
|  |  | CerG1(d18:1/24:0)+H | CerG1 | 1.836 | 0.006 |
|  |  | SM(d38:4)+H | SM | 1.843 | < 0.001 |
|  |  | PS(20:3/22:4)-H | PS | 1.855 | < 0.001 |
|  |  | PS(19:0/22:6)-H | PS | 1.857 | < 0.001 |
|  |  | TG(16:0/16:0/18:2)+NH4 | TG | 1.867 | < 0.001 |
|  |  | CerG1(d18:1/23:0)+H | CerG1 | 1.873 | < 0.001 |
|  |  | PE(18:1/22:6)-H | PE | 1.876 | < 0.001 |
|  |  | PE(18:0p/22:0)-H | PE | 1.878 | < 0.001 |
|  |  | PC(18:1/21:1)+H | PC | 1.882 | < 0.001 |
|  |  | Cer(d18:1/23:1)+H | Cer | 1.886 | < 0.001 |
|  |  | CerG1(d18:1/20:0)+H | CerG1 | 1.886 | < 0.001 |
|  |  | PS(18:0/22:6)-H | PS | 1.889 | < 0.001 |
|  |  | PE(18:1p/20:1)+H | PE | 1.898 | < 0.001 |
|  |  | Cer(d18:0/24:1)+H | Cer | 1.901 | < 0.001 |
|  |  | Cer(d18:1/24:1)+H | Cer | 1.914 | 0.001 |
|  |  | Cer(d20:0/18:0)+H | Cer | 1.920 | < 0.001 |
|  |  | Cer(d20:0+pO/26:0)+HCOO | Cer | 1.930 | < 0.001 |
|  |  | Cer(d30:0)+H | Cer | 1.945 | < 0.001 |
|  |  | TG(18:1/18:2/24:0)+NH4 | TG | 1.983 | < 0.001 |
|  |  | CerG1(d18:1/26:0)+H | CerG1 | 1.987 | < 0.001 |
|  |  | CerG1(d18:1/24:0+O)+H | CerG1 | 1.992 | < 0.001 |
|  |  | TG(16:0/16:0/22:4)+NH4 | TG | 1.993 | < 0.001 |
|  |  | LPE(20:2)-H | LPE | 1.994 | < 0.001 |
|  |  | Cer(d30:3)+H | Cer | 2.013 | 0.003 |
|  |  | Cer(d34:3)+H | Cer | 2.014 | < 0.001 |
|  |  | CerG1(d18:1/24:2)+H | CerG1 | 2.020 | < 0.001 |
|  |  | Cer(d18:1/24:1)+H | Cer | 2.022 | < 0.001 |
|  |  | Cer(d18:0/22:0)+H | Cer | 2.029 | < 0.001 |
|  |  | CerG1(d18:0/24:0+O)+H | CerG1 | 2.031 | < 0.001 |
|  |  | CerG1(d18:1/22:0)+H | CerG1 | 2.032 | < 0.001 |
|  |  | Cer(d18:0/20:0+O)+H | Cer | 2.041 | < 0.001 |
|  |  | PS(38:4p)-H | PS | 2.051 | < 0.001 |
|  |  | CerG1(d18:1/26:1)+H | CerG1 | 2.053 | < 0.001 |
|  |  | CerG1(d18:0/25:0+O)+H | CerG1 | 2.058 | < 0.001 |
|  |  | PS(42:7p)-H | PS | 2.059 | < 0.001 |
|  |  | Cer(d18:0/24:0)+H | Cer | 2.061 | < 0.001 |
|  |  | CerG1(d18:0/22:0+O)+H | CerG1 | 2.081 | < 0.001 |
|  |  | CerG1(d18:0/24:1)+H | CerG1 | 2.082 | < 0.001 |
|  |  | CerG1(d18:1/24:1)+H | CerG1 | 2.086 | < 0.001 |
|  |  | CerG1(d18:0/24:0)+H | CerG1 | 2.093 | < 0.001 |
|  |  | CerG1(d18:0/23:0)+H | CerG1 | 2.094 | < 0.001 |
|  |  | CerG1(d18:1/26:0+O)+H | CerG1 | 2.099 | < 0.001 |
|  |  | Cer(d18:1/24:1)+H | Cer | 2.110 | < 0.001 |
|  |  | Cer(d18:0/23:0+O)+H | Cer | 2.111 | < 0.001 |
|  |  | CerG1(d18:0/22:0+O)+H | CerG1 | 2.117 | < 0.001 |
|  |  | Cer(d18:1/24:0)+H | Cer | 2.121 | < 0.001 |
|  |  | Cer(d18:1/22:0)+H | Cer | 2.122 | < 0.001 |
|  |  | TG(19:0/18:1/18:1)+NH4 | TG | 2.133 | < 0.001 |
|  |  | PS(18:0p/22:6)-H | PS | 2.137 | < 0.001 |
|  |  | CerG1(d18:0+pO/24:0)+H | CerG1 | 2.155 | < 0.001 |
|  |  | CerG1(d18:0+pO/24:2)+H | CerG1 | 2.156 | < 0.001 |
|  |  | CerG1(d18:1/24:0)+H | CerG1 | 2.173 | < 0.001 |
|  |  | TG(20:0/18:1/18:1)+NH4 | TG | 2.179 | < 0.001 |
|  |  | DG(16:0/18:2)+NH4 | DG | 2.202 | < 0.001 |
|  |  | CerG1(d18:0+pO/40:2)+H | CerG1 | 2.208 | < 0.001 |
|  |  | PS(22:5/22:6)-H | PS | 2.217 | < 0.001 |
|  |  | CerG1(d18:1/24:0+O)+H | CerG1 | 2.223 | < 0.001 |
|  |  | Cer(d18:0/24:0+O)+H | Cer | 2.232 | < 0.001 |
|  |  | PE(18:1p/22:6)-H | PE | 2.240 | < 0.001 |
|  |  | PS(18:1/22:6)-H | PS | 2.268 | < 0.001 |
|  |  | CerG1(d18:0/22:0)+H | CerG1 | 2.284 | < 0.001 |
|  |  | PS(20:2/22:6)-H | PS | 2.292 | < 0.001 |
|  |  | TG(18:1/18:1/21:0)+NH4 | TG | 2.298 | < 0.001 |
|  |  | TG(26:0/18:1/18:1)+NH4 | TG | 2.302 | < 0.001 |
|  |  | CerG1(d18:1/44:0EO)+H | CerG1 | 2.307 | < 0.001 |
|  |  | PS(44:11)-H | PS | 2.308 | < 0.001 |
|  |  | SM(d38:5)+H | SM | 2.334 | < 0.001 |
|  |  | CerG1(d18:1/22:0+O)+H | CerG1 | 2.362 | < 0.001 |
|  |  | PS(20:3/22:6)-H | PS | 2.385 | < 0.001 |
|  |  | PE(18:0p/22:3)+H | PE | 2.392 | < 0.001 |
|  |  | Cer(d18:0/22:0+O)+H | Cer | 2.401 | < 0.001 |
|  |  | LPC(24:0)+H | LPC | 2.441 | < 0.001 |
|  |  | Cer(d18:2/16:0)+HCOO | Cer | 2.443 | 0.001 |
|  |  | CerG1(d18:1/22:0)+H | CerG1 | 2.460 | < 0.001 |
|  |  | TG(16:0/18:1/18:1)+NH4 | TG | 2.467 | < 0.001 |
|  |  | PS(20:2/22:4)-H | PS | 2.548 | < 0.001 |
|  |  | TG(18:1/18:1/24:0)+NH4 | TG | 2.686 | < 0.001 |
|  |  | PS(22:6/22:6)-H | PS | 2.688 | < 0.001 |
|  |  | PS(20:4/22:6)-H | PS | 2.713 | < 0.001 |
|  |  | PS(40:8p)-H | PS | 2.743 | < 0.001 |
|  |  | PS(40:6p)-H | PS | 2.754 | < 0.001 |
|  |  | PS(16:1/18:1)-H | PS | 2.777 | < 0.001 |
|  |  | TG(18:1/18:1/22:0)+NH4 | TG | 2.781 | < 0.001 |
|  |  | TG(16:0/17:1/18:2)+NH4 | TG | 2.843 | < 0.001 |
|  |  | Cer(d22:1)+H | Cer | 2.858 | < 0.001 |
|  |  | TG(20:0/18:1/18:2)+NH4 | TG | 2.895 | < 0.001 |
|  |  | TG(18:1/18:1/23:0)+NH4 | TG | 3.135 | < 0.001 |
|  |  | PS(41:2)-H | PS | 3.144 | < 0.001 |
|  |  | DG(34:1e)+Na | DG | 3.291 | < 0.001 |
|  |  | TG(18:3/18:2/18:2)+NH4 | TG | 3.376 | < 0.001 |
|  |  | PI(18:0/18:1)-H | PI | 3.535 | < 0.001 |
|  |  | TG(16:0/18:1/18:2)+NH4 | TG | 3.562 | < 0.001 |
|  |  | TG(16:0/18:2/18:2)+NH4 | TG | 3.811 | < 0.001 |
|  |  | TG(18:1/18:1/18:3)+NH4 | TG | 4.031 | < 0.001 |
|  |  | TG(17:0/18:1/18:1)+NH4 | TG | 4.237 | < 0.001 |
|  |  | PS(42:7p)-H | PS | 4.785 | < 0.001 |
|  |  | TG(16:0/18:1/20:1)+NH4 | TG | 4.951 | < 0.001 |
|  |  | LPI(18:1)-H | LPI | 5.660 | < 0.001 |
|  |  | TG(56:6)+NH4 | TG | 5.689 | 0.001 |
|  |  | TG(18:1/18:1/18:2)+NH4 | TG | 5.807 | < 0.001 |
|  |  | TG(18:1/18:1/18:1)+NH4 | TG | 6.330 | < 0.001 |
|  | **EA *vs.* Sham** | DG(34:1e)+Na | DG | 0.085 | < 0.001 |
|  |  | LPI(18:0)-H | LPI | 0.186 | < 0.001 |
|  |  | TG(18:1/18:1/18:3)+NH4 | TG | 0.235 | < 0.001 |
|  |  | TG(16:0/16:0/22:4)+NH4 | TG | 0.294 | < 0.001 |
|  |  | TG(20:0/18:1/18:2)+NH4 | TG | 0.359 | 0.024 |
|  |  | TG(16:0/18:1/18:2)+NH4 | TG | 0.372 | < 0.001 |
|  |  | LPS(18:1)-H | LPS | 0.489 | < 0.001 |
|  |  | TG(18:1/18:1/18:1)+NH4 | TG | 0.504 | < 0.001 |
|  |  | CL(22:6/22:6/22:6/20:4)-H | CL | 0.512 | < 0.001 |
|  |  | PS(19:0/22:6)-H | PS | 0.532 | < 0.001 |
|  |  | CL(18:2/20:4/16:0/20:4)-H | CL | 0.557 | < 0.001 |
|  |  | PC(34:1)+H | PC | 0.559 | < 0.001 |
|  |  | SM(d52:2+pO)+H | SM | 0.567 | < 0.001 |
|  |  | PE(16:0/22:6)-H | PE | 0.578 | < 0.001 |
|  |  | LPE(22:5)-H | LPE | 0.601 | 0.001 |
|  |  | CL(22:6/22:6/16:1/20:4)-H | CL | 0.612 | < 0.001 |
|  |  | PS(18:3/20:4)-H | PS | 0.621 | < 0.001 |
|  |  | PE(36:1/22:6)-H | PE | 0.637 | < 0.001 |
|  |  | PS(42:5)-H | PS | 0.638 | < 0.001 |
|  |  | DG(34:2e)+Na | DG | 0.648 | 0.001 |
|  |  | PC(37:0e)+H | PC | 0.649 | < 0.001 |
|  |  | PE(16:0e/20:4)-H | PE | 0.649 | < 0.001 |
|  |  | PE(34:3)+H | PE | 0.660 | < 0.001 |
|  |  | PI(18:0/18:1)-H | PI | 0.665 | < 0.001 |
|  |  | PC(18:1p/18:1)+HCOO | PC | 1.503 | < 0.001 |
|  |  | PC(32:4)+H | PC | 1.504 | < 0.001 |
|  |  | CL(18:1/16:0/16:0/20:0)-H | CL | 1.504 | < 0.001 |
|  |  | PC(25:0/18:1)+HCOO | PC | 1.512 | < 0.001 |
|  |  | TG(56:6)+NH4 | TG | 1.513 | < 0.001 |
|  |  | MGMG(18:1)+HCOO | MGMG | 1.516 | < 0.001 |
|  |  | PC(39:1)+H | PC | 1.517 | < 0.001 |
|  |  | MGDG(18:1/23:0)+HCOO | MGDG | 1.517 | < 0.001 |
|  |  | PC(40:3)+H | PC | 1.527 | < 0.001 |
|  |  | PC(36:1e)+H | PC | 1.531 | < 0.001 |
|  |  | DG(16:0/18:2)+NH4 | DG | 1.532 | 0.018 |
|  |  | PS(18:0/18:1)-H | PS | 1.535 | < 0.001 |
|  |  | PS(36:3)-H | PS | 1.535 | < 0.001 |
|  |  | PC(18:1/23:0)+HCOO | PC | 1.537 | < 0.001 |
|  |  | PC(24:0/18:2)+HCOO | PC | 1.538 | < 0.001 |
|  |  | PC(34:2e)+H | PC | 1.539 | < 0.001 |
|  |  | PC(19:1/18:0)+HCOO | PC | 1.539 | < 0.001 |
|  |  | PC(20:1/18:1)+HCOO | PC | 1.546 | < 0.001 |
|  |  | PC(16:0p/20:0)+HCOO | PC | 1.551 | < 0.001 |
|  |  | PC(14:0/20:4)+HCOO | PC | 1.551 | < 0.001 |
|  |  | SM(d41:2)+H | SM | 1.563 | 0.002 |
|  |  | PC(43:4)+H | PC | 1.565 | < 0.001 |
|  |  | PS(42:7p)-H | PS | 1.578 | < 0.001 |
|  |  | PC(18:0p/18:1)+HCOO | PC | 1.579 | < 0.001 |
|  |  | PI(16:0/22:4)-H | PI | 1.579 | < 0.001 |
|  |  | PC(16:0/24:0)+HCOO | PC | 1.583 | < 0.001 |
|  |  | AcCa(16:1)+H | AcCa | 1.585 | < 0.001 |
|  |  | PC(26:0/18:1)+HCOO | PC | 1.591 | < 0.001 |
|  |  | PC(38:7)+H | PC | 1.595 | < 0.001 |
|  |  | TG(20:0/18:1/18:1)+NH4 | TG | 1.596 | < 0.001 |
|  |  | TG(16:0/18:1/22:0)+NH4 | TG | 1.597 | 0.002 |
|  |  | PG(33:0)-H | PG | 1.602 | < 0.001 |
|  |  | AcCa(18:1)+H | AcCa | 1.602 | < 0.001 |
|  |  | PC(16:1/16:1)+HCOO | PC | 1.602 | < 0.001 |
|  |  | PC(26:0/16:0)+HCOO | PC | 1.603 | < 0.001 |
|  |  | TG(19:0/18:1/18:1)+NH4 | TG | 1.604 | 0.011 |
|  |  | PC(42:4p)+H | PC | 1.607 | < 0.001 |
|  |  | LPC(22:4)+H | LPC | 1.607 | 0.044 |
|  |  | PS(20:3/22:4)-H | PS | 1.609 | < 0.001 |
|  |  | PS(18:0/18:1)-H | PS | 1.610 | < 0.001 |
|  |  | PC(16:0/24:0)+HCOO | PC | 1.612 | < 0.001 |
|  |  | PC(18:0/16:0)+HCOO | PC | 1.615 | < 0.001 |
|  |  | PS(41:2)-H | PS | 1.616 | < 0.001 |
|  |  | LPC(24:0)+H | LPC | 1.616 | 0.001 |
|  |  | PC(16:0/20:5)+HCOO | PC | 1.624 | < 0.001 |
|  |  | CL(22:2/20:4/20:4/18:0)-H | CL | 1.625 | < 0.001 |
|  |  | PC(16:0/22:0)+HCOO | PC | 1.625 | < 0.001 |
|  |  | Cer(d18:1/24:1)+H | Cer | 1.626 | < 0.001 |
|  |  | MGDG(18:1/24:1)+HCOO | MGDG | 1.627 | < 0.001 |
|  |  | CerG1(d18:1/24:0+O)+H | CerG1 | 1.629 | < 0.001 |
|  |  | CerG1(d18:1/24:0)+H | CerG1 | 1.631 | < 0.001 |
|  |  | PC(16:0/22:6)+HCOO | PC | 1.631 | < 0.001 |
|  |  | PG(43:2)-H | PG | 1.631 | < 0.001 |
|  |  | MGDG(16:0/24:0)+HCOO | MGDG | 1.631 | < 0.001 |
|  |  | PC(40:5e)+H | PC | 1.632 | < 0.001 |
|  |  | PC(42:3p)+H | PC | 1.635 | < 0.001 |
|  |  | MGDG(18:1/22:0)+HCOO | MGDG | 1.636 | < 0.001 |
|  |  | TG(18:0/16:0/22:0)+NH4 | TG | 1.645 | 0.003 |
|  |  | Cer(d34:3)+H | Cer | 1.646 | 0.001 |
|  |  | PC(60:5)+H | PC | 1.647 | < 0.001 |
|  |  | PS(36:1p)-H | PS | 1.654 | < 0.001 |
|  |  | PC(18:0p/18:1)+HCOO | PC | 1.657 | < 0.001 |
|  |  | PC(39:0)+H | PC | 1.663 | < 0.001 |
|  |  | PC(40:3)+H | PC | 1.665 | < 0.001 |
|  |  | PC(58:6)+H | PC | 1.677 | < 0.001 |
|  |  | PC(18:0/22:6)+H | PC | 1.683 | < 0.001 |
|  |  | PC(33:1p)+H | PC | 1.683 | < 0.001 |
|  |  | DG(16:0/18:1)+NH4 | DG | 1.684 | < 0.001 |
|  |  | LPI(18:1)-H | LPI | 1.688 | < 0.001 |
|  |  | TG(17:0/18:1/18:1)+NH4 | TG | 1.695 | < 0.001 |
|  |  | PI(18:0/20:3)-H | PI | 1.699 | < 0.001 |
|  |  | PS(36:4)-H | PS | 1.700 | < 0.001 |
|  |  | SQDG(37:0)+HCOO | SQDG | 1.705 | < 0.001 |
|  |  | PC(35:2)+H | PC | 1.707 | < 0.001 |
|  |  | SM(d17:1/18:1)+HCOO | SM | 1.708 | < 0.001 |
|  |  | PC(43:2)+H | PC | 1.718 | < 0.001 |
|  |  | SM(d41:0)+H | SM | 1.721 | < 0.001 |
|  |  | So(d18:0)+H | So | 1.723 | 0.001 |
|  |  | SM(d38:4)+H | SM | 1.728 | < 0.001 |
|  |  | MGMG(16:0)+HCOO | MGMG | 1.743 | < 0.001 |
|  |  | TG(18:0/16:0/24:0)+NH4 | TG | 1.743 | < 0.001 |
|  |  | PC(42:1e)+H | PC | 1.749 | < 0.001 |
|  |  | PC(18:1/22:0)+HCOO | PC | 1.753 | < 0.001 |
|  |  | AcCa(14:0)+H | AcCa | 1.756 | < 0.001 |
|  |  | PS(38:4p)-H | PS | 1.769 | < 0.001 |
|  |  | MGDG(16:0/20:1)+HCOO | MGDG | 1.785 | < 0.001 |
|  |  | TG(18:3/18:2/18:2)+NH4 | TG | 1.790 | < 0.001 |
|  |  | LPS(18:0)-H | LPS | 1.822 | < 0.001 |
|  |  | AcCa(20:0)+H | AcCa | 1.828 | < 0.001 |
|  |  | DG(18:0/20:3)+NH4 | DG | 1.835 | 0.005 |
|  |  | MGMG(18:1)+HCOO | MGMG | 1.841 | < 0.001 |
|  |  | SM(d22:0/18:0)+HCOO | SM | 1.846 | < 0.001 |
|  |  | TG(18:1/18:1/21:1)+NH4 | TG | 1.853 | < 0.001 |
|  |  | PS(38:3p)-H | PS | 1.869 | < 0.001 |
|  |  | PC(35:2)+H | PC | 1.874 | < 0.001 |
|  |  | PC(42:2)+H | PC | 1.884 | < 0.001 |
|  |  | MGDG(18:1/20:1)+HCOO | MGDG | 1.886 | < 0.001 |
|  |  | SM(d22:1/16:0)+HCOO | SM | 1.886 | < 0.001 |
|  |  | PC(44:4)+H | PC | 1.895 | < 0.001 |
|  |  | PG(44:2p)-H | PG | 1.899 | < 0.001 |
|  |  | PI(18:1/18:1)-H | PI | 1.904 | < 0.001 |
|  |  | DG(18:0/22:4)+NH4 | DG | 1.914 | 0.001 |
|  |  | PS(18:1/18:1)-H | PS | 1.924 | 0.294 |
|  |  | DG(18:1/24:0)+NH4 | DG | 1.933 | < 0.001 |
|  |  | CerG1(d18:1/25:1)+H | CerG1 | 1.941 | < 0.001 |
|  |  | PS(36:3p)-H | PS | 1.947 | < 0.001 |
|  |  | PC(23:0e)+H | PC | 1.974 | < 0.001 |
|  |  | PC(40:7)+H | PC | 1.985 | < 0.001 |
|  |  | SM(d35:1)+HCOO | SM | 2.003 | < 0.001 |
|  |  | SM(d42:3)+HCOO | SM | 2.022 | < 0.001 |
|  |  | PC(32:1/20:4)+HCOO | PC | 2.042 | < 0.001 |
|  |  | PC(38:5)+H | PC | 2.087 | < 0.001 |
|  |  | SM(d38:5)+H | SM | 2.123 | < 0.001 |
|  |  | PC(18:0p/20:1)+HCOO | PC | 2.137 | < 0.001 |
|  |  | MGDG(16:0/20:0)+HCOO | MGDG | 2.169 | < 0.001 |
|  |  | PE(36:1/22:6)-H | PE | 2.170 | < 0.001 |
|  |  | SM(d44:2)+H | SM | 2.220 | < 0.001 |
|  |  | MGDG(16:0/18:1)+HCOO | MGDG | 2.222 | < 0.001 |
|  |  | PE(18:1p/22:6)-H | PE | 2.244 | < 0.001 |
|  |  | PC(35:0p)+H | PC | 2.252 | < 0.001 |
|  |  | DG(18:1/18:1)+NH4 | DG | 2.274 | < 0.001 |
|  |  | PS(38:4p)-H | PS | 2.319 | < 0.001 |
|  |  | PC(26:2p)+H | PC | 2.408 | < 0.001 |
|  |  | DG(18:1/24:1)+NH4 | DG | 2.425 | < 0.001 |
|  |  | MGDG(16:0/18:0)+HCOO | MGDG | 2.436 | < 0.001 |
|  |  | MGDG(18:1/18:1)+HCOO | MGDG | 2.459 | < 0.001 |
|  |  | PC(18:0/22:6)+HCOO | PC | 2.537 | < 0.001 |
|  |  | DG(18:0/20:1)+NH4 | DG | 2.600 | < 0.001 |
|  |  | PG(38:1p)-H | PG | 2.725 | < 0.001 |
|  |  | DG(18:1/22:0)+NH4 | DG | 2.804 | < 0.001 |
|  |  | PS(18:1/24:0)-H | PS | 2.957 | < 0.001 |
|  |  | PC(37:1p)+H | PC | 2.985 | < 0.001 |
|  |  | DG(18:1/22:1)+NH4 | DG | 3.136 | < 0.001 |
|  |  | PC(38:2)+H | PC | 3.212 | < 0.001 |
|  |  |  |  |  |  |
| **prefrontal cortex** | **PTSD+Sham *vs.* Sham** | PS(18:0/22:4)-H | PS | 0.259 | <0.001 |
|  |  | PS(22:6/22:6)-H | PS | 0.335 | <0.001 |
|  |  | PS(42:7p)-H | PS | 0.377 | <0.001 |
|  |  | PS(20:2/22:6)-H | PS | 0.379 | <0.001 |
|  |  | PS(41:2)-H | PS | 0.380 | <0.001 |
|  |  | PS(38:4p)-H | PS | 0.383 | <0.001 |
|  |  | PS(42:7p)-H | PS | 0.392 | <0.001 |
|  |  | PS(20:2/22:4)-H | PS | 0.401 | <0.001 |
|  |  | PS(20:2/22:5)-H | PS | 0.410 | <0.001 |
|  |  | PS(37:1)-H | PS | 0.410 | <0.001 |
|  |  | PS(43:1)-H | PS | 0.422 | <0.001 |
|  |  | PS(42:3)-H | PS | 0.432 | <0.001 |
|  |  | LPG(16:0)-H | LPG | 0.434 | <0.001 |
|  |  | PS(40:6p)-H | PS | 0.440 | <0.001 |
|  |  | PS(18:1/22:6)-H | PS | 0.441 | <0.001 |
|  |  | PS(41:3)-H | PS | 0.442 | <0.001 |
|  |  | PS(22:5/22:6)-H | PS | 0.451 | <0.001 |
|  |  | PS(42:5)-H | PS | 0.453 | <0.001 |
|  |  | PS(20:3/22:4)-H | PS | 0.458 | <0.001 |
|  |  | PS(36:3p)-H | PS | 0.458 | <0.001 |
|  |  | PS(38:4p)-H | PS | 0.459 | <0.001 |
|  |  | PS(19:0/22:6)-H | PS | 0.462 | <0.001 |
|  |  | PE(16:0e/20:4)-H | PE | 0.463 | <0.001 |
|  |  | PS(18:1/18:1)-H | PS | 0.463 | <0.001 |
|  |  | PS(18:1/20:2)-H | PS | 0.465 | <0.001 |
|  |  | PS(20:4/22:6)-H | PS | 0.467 | <0.001 |
|  |  | DG(16:0/18:2)+NH4 | DG | 0.471 | <0.001 |
|  |  | PS(18:2/22:6)-H | PS | 0.473 | <0.001 |
|  |  | PS(16:0/18:1)-H | PS | 0.473 | <0.001 |
|  |  | PS(44:11)-H | PS | 0.478 | <0.001 |
|  |  | PS(22:6/22:6)-H | PS | 0.482 | <0.001 |
|  |  | PS(40:8p)-H | PS | 0.487 | <0.001 |
|  |  | PS(20:2/20:3)-H | PS | 0.488 | <0.001 |
|  |  | PS(20:3/22:6)-H | PS | 0.489 | <0.001 |
|  |  | PS(16:0/22:6)-H | PS | 0.491 | <0.001 |
|  |  | PS(18:1/18:1)-H | PS | 0.494 | <0.001 |
|  |  | Cer(d22:1)+H | Cer | 0.495 | <0.001 |
|  |  | PS(18:1/22:2)-H | PS | 0.496 | <0.001 |
|  |  | PS(18:0/22:6)-H | PS | 0.500 | <0.001 |
|  |  | PS(18:0/20:4)-H | PS | 0.500 | <0.001 |
|  |  | PS(36:1p)-H | PS | 0.505 | <0.001 |
|  |  | PS(20:4/22:6)-H | PS | 0.505 | <0.001 |
|  |  | PS(18:1/18:1)+H | PS | 0.506 | <0.001 |
|  |  | PS(38:3p)-H | PS | 0.508 | <0.001 |
|  |  | PS(18:3/20:4)-H | PS | 0.512 | <0.001 |
|  |  | PC(32:0)+H | PC | 0.514 | 0.028 |
|  |  | AcCa(18:2)+H | AcCa | 0.524 | 0.009 |
|  |  | LPC(24:1)+HCOO | LPC | 0.524 | <0.001 |
|  |  | PS(20:4/22:6)-H | PS | 0.525 | <0.001 |
|  |  | PS(34:2)-H | PS | 0.526 | <0.001 |
|  |  | PS(18:0/22:5)-H | PS | 0.534 | <0.001 |
|  |  | PS(16:1/18:1)-H | PS | 0.542 | 0.001 |
|  |  | Cer(d20:1)+H | Cer | 0.544 | <0.001 |
|  |  | PS(18:1/22:6)-H | PS | 0.547 | <0.001 |
|  |  | LPG(20:4)-H | LPG | 0.549 | <0.001 |
|  |  | PS(18:0/22:6)-H | PS | 0.551 | <0.001 |
|  |  | PS(16:0/20:4)-H | PS | 0.562 | <0.001 |
|  |  | AcCa(20:4)+H | AcCa | 0.562 | 0.001 |
|  |  | PC(18:0/16:0)+HCOO | PC | 0.563 | 0.042 |
|  |  | LPS(22:6)-H | LPS | 0.564 | <0.001 |
|  |  | PS(18:1/20:4)-H | PS | 0.565 | <0.001 |
|  |  | PS(22:5/22:6)-H | PS | 0.565 | <0.001 |
|  |  | PS(22:4/22:6)-H | PS | 0.569 | <0.001 |
|  |  | AcCa(22:6)+H | AcCa | 0.571 | 0.001 |
|  |  | LPC(16:1p)+H | LPC | 0.582 | 0.046 |
|  |  | PG(42:6)-H | PG | 0.589 | 0.001 |
|  |  | PE(16:0p/20:5)-H | PE | 0.589 | 0.002 |
|  |  | PS(18:3/22:6)-H | PS | 0.593 | <0.001 |
|  |  | PS(43:5)-H | PS | 0.598 | <0.001 |
|  |  | Cer(d20:0+pO/26:0)+HCOO | Cer | 0.599 | 0.011 |
|  |  | MGDG(10:4/22:6)+HCOO | MGDG | 0.601 | 0.002 |
|  |  | PC(18:1/22:6)+H | PC | 0.604 | 0.027 |
|  |  | Cer(d32:0+pO)+H | Cer | 0.611 | 0.001 |
|  |  | CL(83:9)-H | CL | 0.613 | 0.032 |
|  |  | PC(19:1/18:0)+HCOO | PC | 0.613 | 0.012 |
|  |  | PS(18:0/18:1)-H | PS | 0.618 | <0.001 |
|  |  | PS(18:0/22:6)-H | PS | 0.619 | <0.001 |
|  |  | PC(18:1/18:2)+HCOO | PC | 0.626 | 0.023 |
|  |  | PS(41:6)-H | PS | 0.628 | 0.002 |
|  |  | PS(36:3)-H | PS | 0.633 | <0.001 |
|  |  | Cer(d42:4)+H | Cer | 0.635 | <0.001 |
|  |  | LPC(15:0)+H | LPC | 0.639 | 0.026 |
|  |  | PS(36:4)-H | PS | 0.640 | <0.001 |
|  |  | PE(18:1p/22:6)-H | PE | 0.641 | <0.001 |
|  |  | CL(82:18)-H | CL | 0.643 | 0.002 |
|  |  | PS(45:6)-H | PS | 0.644 | 0.001 |
|  |  | CL(18:1/20:3/18:1/22:6)-H | CL | 0.652 | 0.005 |
|  |  | PE(18:2p/20:4)-H | PE | 0.654 | 0.008 |
|  |  | PG(40:5)+NH4 | PG | 0.658 | 0.001 |
|  |  | Cer(d32:3)+H | Cer | 0.663 | 0.013 |
|  |  | MGDG(16:0/16:3)+HCOO | MGDG | 0.664 | 0.004 |
|  |  | LPC(16:0)+H | LPC | 0.666 | 0.009 |
|  |  | Co(Q9)+NH4 | Co | 0.669 | 0.001 |
|  |  | CerG1(d18:1/24:0+O)+H | CerG1 | 1.500 | 0.002 |
|  |  | SM(d41:0)+H | SM | 1.500 | 0.028 |
|  |  | CerG1(d18:0/22:0)+H | CerG1 | 1.510 | 0.049 |
|  |  | TG(16:0/18:1/20:4)+NH4 | TG | 1.526 | <0.001 |
|  |  | TG(16:0/16:0/20:4)+NH4 | TG | 1.538 | 0.001 |
|  |  | DG(16:0/18:1)+NH4 | DG | 1.539 | <0.001 |
|  |  | SM(d22:0/20:1)+HCOO | SM | 1.541 | <0.001 |
|  |  | SM(d43:1)+H | SM | 1.548 | 0.001 |
|  |  | Cer(d18:0/24:1)+H | Cer | 1.566 | 0.040 |
|  |  | WE(23:2)+NH4 | WE | 1.584 | <0.001 |
|  |  | LPC(14:0)+HCOO | LPC | 1.587 | 0.001 |
|  |  | DG(18:1/22:0)+NH4 | DG | 1.590 | 0.005 |
|  |  | TG(40:4p)+NH4 | TG | 1.596 | 0.018 |
|  |  | PE(38:2p)-H | PE | 1.597 | 0.016 |
|  |  | DG(32:1e)+Na | DG | 1.641 | <0.001 |
|  |  | PC(37:1p)+H | PC | 1.650 | 0.002 |
|  |  | DG(34:1e)+Na | DG | 1.652 | 0.001 |
|  |  | DG(32:0e)+Na | DG | 1.681 | 0.011 |
|  |  | DG(34:1e)+Na | DG | 1.728 | 0.001 |
|  |  | MGMG(18:1)+HCOO | MGMG | 1.728 | 0.003 |
|  |  | PC(42:1e)+H | PC | 1.745 | 0.001 |
|  |  | LPI(18:1)-H | LPI | 1.768 | <0.001 |
|  |  | DG(18:1/24:1)+NH4 | DG | 1.770 | 0.001 |
|  |  | DG(18:0/20:3)+NH4 | DG | 1.795 | <0.001 |
|  |  | DG(18:1/24:0)+NH4 | DG | 1.807 | 0.001 |
|  |  | SM(d18:1/27:0)+H | SM | 1.840 | 0.026 |
|  |  | DG(18:0/18:1)+NH4 | DG | 1.860 | 0.003 |
|  |  | TG(16:0/20:4/20:4)+NH4 | TG | 1.895 | <0.001 |
|  |  | DG(18:0/20:1)+NH4 | DG | 1.910 | 0.002 |
|  |  | SM(d41:2)+H | SM | 1.946 | 0.001 |
|  |  | LPE(20:1)-H | LPE | 1.976 | 0.003 |
|  |  | WE(21:1)+NH4 | WE | 2.024 | <0.001 |
|  |  | DG(18:1/22:1)+NH4 | DG | 2.047 | 0.001 |
|  |  | LPE(20:3)-H | LPE | 2.076 | <0.001 |
|  |  | LPE(20:2)-H | LPE | 2.242 | 0.001 |
|  |  | PC(33:0)+H | PC | 3.241 | 0.021 |
|  |  | PC(32:0)+H | PC | 10.842 | 0.001 |
|  | **PTSD+Sham *vs.* PTSD+EA** | LPG(16:0)-H | LPG | 0.345 | <0.001 |
|  |  | LPG(20:4)-H | LPG | 0.367 | <0.001 |
|  |  | Co(Q9)+NH4 | Co | 0.486 | <0.001 |
|  |  | DG(16:0/18:2)+NH4 | DG | 0.563 | 0.032 |
|  |  | TG(16:0/18:1/21:0)+NH4 | TG | 0.612 | 0.012 |
|  |  | PC(38:7)+H | PC | 0.613 | 0.001 |
|  |  | PG(40:5)+NH4 | PG | 0.617 | 0.019 |
|  |  | Cer(d20:1)+H | Cer | 0.631 | 0.022 |
|  |  | PS(18:1/18:1)+H | PS | 0.638 | <0.001 |
|  |  | LPC(16:1p)+H | LPC | 0.646 | 0.026 |
|  |  | PC(16:1/16:1)+HCOO | PC | 0.659 | 0.001 |
|  |  | PS(20:2/22:5)-H | PS | 1.542 | <0.001 |
|  |  | TG(18:1/18:1/18:2)+NH4 | TG | 1.550 | 0.012 |
|  |  | DG(32:0e)+Na | DG | 1.555 | 0.029 |
|  |  | PS(34:2)-H | PS | 1.573 | 0.008 |
|  |  | TG(16:0/18:2/18:2)+NH4 | TG | 1.608 | 0.021 |
|  |  | PS(42:7p)-H | PS | 1.704 | <0.001 |
|  |  | TG(18:1/18:1/18:1)+NH4 | TG | 1.747 | <0.001 |
|  |  | PS(18:0/22:4)-H | PS | 2.252 | <0.001 |
|  |  | LPE(20:2)-H | LPE | 2.849 | <0.001 |
|  |  | LPE(20:3)-H | LPE | 3.033 | <0.001 |
|  | **EA *vs.* Sham** | PS(38:4p)-H | PS | 0.368 | <0.001 |
|  |  | PS(42:7p)-H | PS | 0.403 | <0.001 |
|  |  | PS(16:0/20:4)-H | PS | 0.445 | <0.001 |
|  |  | PS(41:3)-H | PS | 0.461 | <0.001 |
|  |  | PS(20:4/22:6)-H | PS | 0.471 | <0.001 |
|  |  | PC(32:0)+H | PC | 0.474 | 0.018 |
|  |  | PS(18:0/22:4)-H | PS | 0.493 | <0.001 |
|  |  | PS(22:6/22:6)-H | PS | 0.506 | <0.001 |
|  |  | AcCa(18:2)+H | AcCa | 0.506 | 0.007 |
|  |  | PS(20:4/22:6)-H | PS | 0.518 | <0.001 |
|  |  | PS(22:5/22:6)-H | PS | 0.524 | <0.001 |
|  |  | CL(18:2/20:4/16:0/20:4)-H | CL | 0.528 | 0.004 |
|  |  | PA(35:1)-H | PA | 0.536 | <0.001 |
|  |  | PS(18:1/18:1)-H | PS | 0.550 | <0.001 |
|  |  | PS(20:4/22:6)-H | PS | 0.553 | <0.001 |
|  |  | DG(34:2e)+Na | DG | 0.554 | <0.001 |
|  |  | PS(41:2)-H | PS | 0.555 | <0.001 |
|  |  | PS(41:6)-H | PS | 0.582 | <0.001 |
|  |  | PS(20:2/22:4)-H | PS | 0.584 | <0.001 |
|  |  | PS(37:1)-H | PS | 0.589 | <0.001 |
|  |  | LPS(22:6)-H | LPS | 0.599 | 0.002 |
|  |  | PE(18:1p/22:5)-H | PE | 0.599 | 0.001 |
|  |  | PS(18:0p/22:6)-H | PS | 0.609 | <0.001 |
|  |  | PS(22:6/22:6)-H | PS | 0.621 | <0.001 |
|  |  | PS(18:3/20:4)-H | PS | 0.625 | <0.001 |
|  |  | PE(16:0e/20:4)-H | PE | 0.629 | <0.001 |
|  |  | PS(19:0/22:6)-H | PS | 0.639 | <0.001 |
|  |  | LPS(18:1)-H | LPS | 0.656 | 0.001 |
|  |  | DG(34:1e)+Na | DG | 0.658 | 0.048 |
|  |  | PS(16:0/22:6)-H | PS | 0.660 | <0.001 |
|  |  | PE(18:0p/23:1)+H | PE | 1.505 | 0.018 |
|  |  | CerG1(d18:1/24:0+O)+H | CerG1 | 1.506 | 0.003 |
|  |  | PE(54:2)-H | PE | 1.507 | 0.017 |
|  |  | PE(16:0/16:0)-H | PE | 1.508 | <0.001 |
|  |  | PC(42:10)+H | PC | 1.511 | 0.015 |
|  |  | PC(38:4)+H | PC | 1.512 | <0.001 |
|  |  | PC(33:0p)+H | PC | 1.513 | 0.001 |
|  |  | LPI(18:0)-H | LPI | 1.517 | 0.001 |
|  |  | Cer(d16:0/28:1)+HCOO | Cer | 1.518 | 0.048 |
|  |  | PC(17:1/18:1)+HCOO | PC | 1.520 | <0.001 |
|  |  | phSM(d39:6+hO)+HCOO | phSM | 1.522 | <0.001 |
|  |  | TG(16:0/18:1/20:1)+NH4 | TG | 1.523 | 0.036 |
|  |  | LPC(18:1p)+H | LPC | 1.523 | 0.026 |
|  |  | MGDG(18:0/20:1)+HCOO | MGDG | 1.524 | 0.028 |
|  |  | TG(16:0/17:1/18:1)+NH4 | TG | 1.525 | 0.027 |
|  |  | CerG1(d18:1/18:0+O)+H | CerG1 | 1.528 | <0.001 |
|  |  | CerG1(d18:0/24:0+O)+H | CerG1 | 1.530 | 0.007 |
|  |  | LPC(18:1)+HCOO | LPC | 1.532 | <0.001 |
|  |  | CerG1(d18:1/22:1)+H | CerG1 | 1.535 | 0.006 |
|  |  | CerG1(d18:1/23:0+O)+H | CerG1 | 1.536 | 0.011 |
|  |  | CerG1(d18:1/22:0+O)+H | CerG1 | 1.546 | 0.007 |
|  |  | CerG1(d18:1/22:1)+H | CerG1 | 1.547 | 0.001 |
|  |  | TG(16:0/14:0/14:0)+NH4 | TG | 1.548 | 0.010 |
|  |  | CerG1(d18:1/24:2)+H | CerG1 | 1.549 | 0.002 |
|  |  | PC(44:4)+H | PC | 1.549 | <0.001 |
|  |  | PC(19:4e)+H | PC | 1.550 | 0.007 |
|  |  | PE(18:0p/24:1)-H | PE | 1.550 | 0.003 |
|  |  | PE(38:2p)-H | PE | 1.552 | 0.001 |
|  |  | PC(35:2)+H | PC | 1.552 | 0.001 |
|  |  | PE(18:0/22:5)-H | PE | 1.552 | 0.004 |
|  |  | TG(16:0e/18:0/18:1)+NH4 | TG | 1.554 | 0.029 |
|  |  | CerG1(d18:1/20:1)+H | CerG1 | 1.560 | 0.001 |
|  |  | Cer(d18:2/22:1)+H | Cer | 1.560 | 0.001 |
|  |  | CerG1(d18:0+pO/25:2)+H | CerG1 | 1.561 | 0.010 |
|  |  | CL(22:6/20:4/16:1/20:4)-H | CL | 1.561 | 0.012 |
|  |  | CerG1(d18:1/26:2)+H | CerG1 | 1.565 | 0.003 |
|  |  | PC(42:8)+H | PC | 1.570 | <0.001 |
|  |  | CerG1(d18:1/25:2)+H | CerG1 | 1.571 | 0.008 |
|  |  | PG(44:2p)-H | PG | 1.572 | 0.017 |
|  |  | SM(d42:3)+HCOO | SM | 1.578 | 0.001 |
|  |  | Cer(d18:1/22:0+O)+H | Cer | 1.579 | 0.002 |
|  |  | PC(42:2)+H | PC | 1.582 | 0.001 |
|  |  | CerG1(d18:0/22:0+O)+H | CerG1 | 1.583 | 0.025 |
|  |  | PC(16:0/16:0)+HCOO | PC | 1.586 | <0.001 |
|  |  | MGMG(16:0)+HCOO | MGMG | 1.587 | 0.002 |
|  |  | CL(18:1/16:0/16:1/20:4)-H | CL | 1.587 | 0.002 |
|  |  | DG(16:0/18:1)+NH4 | DG | 1.590 | 0.014 |
|  |  | LPE(20:3)-H | LPE | 1.595 | 0.017 |
|  |  | PC(20:0e/16:0)+HCOO | PC | 1.595 | 0.031 |
|  |  | SM(d41:0)+H | SM | 1.597 | 0.014 |
|  |  | GM1(d36:1)-H | GM1 | 1.598 | 0.002 |
|  |  | Cer(d18:1/21:1)+H | Cer | 1.598 | 0.030 |
|  |  | SM(d35:1)+HCOO | SM | 1.600 | 0.024 |
|  |  | SM(d41:1)+HCOO | SM | 1.601 | 0.037 |
|  |  | PE(18:0/18:1)-H | PE | 1.602 | <0.001 |
|  |  | PC(41:0)+H | PC | 1.604 | 0.016 |
|  |  | Cer(d18:1/24:1)+H | Cer | 1.605 | <0.001 |
|  |  | PC(35:0p)+H | PC | 1.609 | 0.003 |
|  |  | SM(d45:6)+H | SM | 1.609 | 0.001 |
|  |  | Cer(d18:1/24:1)+H | Cer | 1.612 | 0.002 |
|  |  | PG(38:1p)-H | PG | 1.614 | <0.001 |
|  |  | PG(33:0)-H | PG | 1.616 | 0.001 |
|  |  | CerG1(d18:2/24:0+O)+H | CerG1 | 1.619 | 0.008 |
|  |  | TG(16:0/18:1/21:0)+NH4 | TG | 1.619 | 0.043 |
|  |  | CerG1(d18:2/23:1)+H | CerG1 | 1.627 | 0.023 |
|  |  | PG(20:4/22:6)-H | PG | 1.628 | <0.001 |
|  |  | TG(16:0/10:0/18:1)+NH4 | TG | 1.635 | 0.024 |
|  |  | PC(60:5)+H | PC | 1.636 | <0.001 |
|  |  | PE(16:0p/18:1)+H | PE | 1.637 | <0.001 |
|  |  | CerG1(d18:0/24:1)+H | CerG1 | 1.639 | 0.011 |
|  |  | PC(16:0/24:0)+HCOO | PC | 1.639 | 0.001 |
|  |  | AcCa(14:0)+H | AcCa | 1.644 | <0.001 |
|  |  | SM(d17:1/18:1)+HCOO | SM | 1.645 | 0.009 |
|  |  | PC(20:1/18:1)+HCOO | PC | 1.646 | <0.001 |
|  |  | CerG1(d18:1/22:0+O)+H | CerG1 | 1.654 | 0.002 |
|  |  | CerG1(d18:1/25:0)+H | CerG1 | 1.655 | 0.002 |
|  |  | CerG1(d18:2/23:1)+H | CerG1 | 1.657 | 0.009 |
|  |  | CerG1(d18:1/23:1)+H | CerG1 | 1.660 | 0.019 |
|  |  | PI(18:1/18:1)-H | PI | 1.665 | <0.001 |
|  |  | PS(18:0/18:1)-H | PS | 1.667 | 0.000 |
|  |  | SM(d22:0/20:1)+HCOO | SM | 1.668 | 0.040 |
|  |  | CL(18:1/16:0/16:0/20:0)-H | CL | 1.670 | <0.001 |
|  |  | PE(36:0)+H | PE | 1.671 | <0.001 |
|  |  | PC(16:0p/20:0)+HCOO | PC | 1.675 | 0.001 |
|  |  | PG(16:0/22:6)-H | PG | 1.676 | <0.001 |
|  |  | Cer(d18:0/20:0+O)+H | Cer | 1.677 | 0.002 |
|  |  | MGMG(18:1)+HCOO | MGMG | 1.677 | <0.001 |
|  |  | Cer(d26:0+pO)+H | Cer | 1.679 | 0.012 |
|  |  | PC(38:1e)+H | PC | 1.684 | 0.005 |
|  |  | Cer(d36:3+O)+H | Cer | 1.685 | 0.013 |
|  |  | Cer(d18:1/24:0+O)+H | Cer | 1.686 | 0.001 |
|  |  | PC(50:6)+H | PC | 1.689 | 0.040 |
|  |  | SM(d18:0/18:1)+HCOO | SM | 1.695 | 0.005 |
|  |  | SM(d38:5)+H | SM | 1.696 | <0.001 |
|  |  | PC(40:3)+H | PC | 1.698 | 0.011 |
|  |  | PE(18:1/23:0)-H | PE | 1.704 | 0.001 |
|  |  | CerG1(d18:1/24:1)+H | CerG1 | 1.707 | 0.003 |
|  |  | PC(40:3)+H | PC | 1.708 | <0.001 |
|  |  | MGDG(18:1/18:1)+HCOO | MGDG | 1.712 | 0.001 |
|  |  | CerG1(d18:1/26:0+O)+H | CerG1 | 1.713 | <0.001 |
|  |  | PC(46:5)+H | PC | 1.714 | 0.002 |
|  |  | Cer(d18:0/22:0+O)+H | Cer | 1.715 | 0.004 |
|  |  | Cer(d18:1/25:2)+H | Cer | 1.716 | 0.027 |
|  |  | CerG1(d18:1/20:0)+H | CerG1 | 1.716 | 0.002 |
|  |  | PC(18:0p/20:1)+HCOO | PC | 1.722 | <0.001 |
|  |  | CerG1(d18:2/24:1)+H | CerG1 | 1.735 | 0.001 |
|  |  | TG(16:0/16:1/16:1)+NH4 | TG | 1.744 | 0.034 |
|  |  | Cer(d18:0/22:0)+H | Cer | 1.748 | 0.014 |
|  |  | TG(18:1/18:1/21:1)+NH4 | TG | 1.751 | 0.013 |
|  |  | MGDG(16:0/18:1)+HCOO | MGDG | 1.752 | <0.001 |
|  |  | DG(16:0/18:2)+NH4 | DG | 1.752 | <0.001 |
|  |  | Cer(d34:3)+H | Cer | 1.760 | 0.012 |
|  |  | CerG1(d43:4)+H | CerG1 | 1.764 | <0.001 |
|  |  | Cer(d18:0/24:0+O)+H | Cer | 1.764 | 0.001 |
|  |  | LPI(20:4)-H | LPI | 1.767 | <0.001 |
|  |  | CerG1(d18:1/23:0)+H | CerG1 | 1.769 | 0.001 |
|  |  | PC(16:0/18:1)+HCOO | PC | 1.776 | <0.001 |
|  |  | PE(18:0p/22:3)+H | PE | 1.778 | <0.001 |
|  |  | CerG1(d18:1/25:1)+H | CerG1 | 1.782 | 0.008 |
|  |  | PC(38:2e)+H | PC | 1.784 | 0.001 |
|  |  | CerG1(d18:0/24:0)+H | CerG1 | 1.785 | 0.001 |
|  |  | SM(d42:2)+HCOO | SM | 1.788 | 0.006 |
|  |  | Cer(d18:1/24:1)+H | Cer | 1.789 | 0.001 |
|  |  | MGDG(18:3/18:3)+HCOO | MGDG | 1.790 | 0.014 |
|  |  | TG(18:0/20:4/20:4)+NH4 | TG | 1.798 | 0.001 |
|  |  | Cer(d18:1/23:0+O)+H | Cer | 1.801 | 0.010 |
|  |  | CerG1(d18:1/24:1)+H | CerG1 | 1.802 | <0.001 |
|  |  | CerG1(d40:0+pO+O)+H | CerG1 | 1.804 | 0.001 |
|  |  | PC(41:4)+H | PC | 1.807 | 0.006 |
|  |  | PC(42:3)+H | PC | 1.808 | <0.001 |
|  |  | CerG1(d42:4)+H | CerG1 | 1.814 | <0.001 |
|  |  | PE(36:1p)-H | PE | 1.818 | 0.005 |
|  |  | LPE(20:2)-H | LPE | 1.821 | 0.017 |
|  |  | CerG1(d18:0/22:0+O)+H | CerG1 | 1.827 | 0.009 |
|  |  | PC(16:0e/24:0)+HCOO | PC | 1.833 | 0.035 |
|  |  | CerG1(d18:0+pO/24:0)+H | CerG1 | 1.837 | 0.001 |
|  |  | Cer(d18:1/25:1)+H | Cer | 1.838 | 0.009 |
|  |  | CerG1(d18:0+pO/26:2)+H | CerG1 | 1.842 | 0.002 |
|  |  | PE(42:2)-H | PE | 1.855 | 0.000 |
|  |  | PC(46:4)+H | PC | 1.861 | 0.012 |
|  |  | PC(43:2)+H | PC | 1.875 | <0.001 |
|  |  | CerG1(d18:1/24:0)+H | CerG1 | 1.889 | <0.001 |
|  |  | SM(d44:2)+H | SM | 1.890 | 0.001 |
|  |  | SM(d22:1/16:0)+HCOO | SM | 1.898 | 0.022 |
|  |  | CL(18:1/16:0/18:1/22:6)-H | CL | 1.899 | <0.001 |
|  |  | SM(d43:1)+H | SM | 1.904 | 0.001 |
|  |  | Cer(d18:2/24:0+O)+H | Cer | 1.904 | 0.023 |
|  |  | Cer(d18:0/23:0+O)+H | Cer | 1.906 | 0.005 |
|  |  | TG(40:4p)+NH4 | TG | 1.912 | 0.007 |
|  |  | PC(18:0/22:6)+HCOO | PC | 1.912 | <0.001 |
|  |  | Cer(d18:1/24:0)+H | Cer | 1.913 | <0.001 |
|  |  | CerG1(d18:0+pO/40:2)+H | CerG1 | 1.919 | 0.001 |
|  |  | PC(40:7)+H | PC | 1.919 | <0.001 |
|  |  | PC(18:0p/18:1)+HCOO | PC | 1.935 | <0.001 |
|  |  | FA(20:4)-H | FA | 1.947 | <0.001 |
|  |  | CerG1(d18:0/22:0)+H | CerG1 | 1.947 | 0.003 |
|  |  | CerG1(d18:1/21:0+O)+H | CerG1 | 1.953 | 0.005 |
|  |  | PE(36:1/18:1)-H | PE | 1.974 | 0.015 |
|  |  | MGMG(18:1)+HCOO | MGMG | 1.977 | <0.001 |
|  |  | SM(d41:2)+H | SM | 1.998 | 0.009 |
|  |  | SM(d38:4)+H | SM | 2.011 | <0.001 |
|  |  | CerG1(d18:1/26:1)+H | CerG1 | 2.019 | 0.002 |
|  |  | PC(18:1/21:1)+H | PC | 2.023 | <0.001 |
|  |  | PC(56:5)+H | PC | 2.027 | <0.001 |
|  |  | PC(39:1)+H | PC | 2.043 | <0.001 |
|  |  | CerG1(d18:1/24:0)+H | CerG1 | 2.044 | 0.001 |
|  |  | CerG1(d18:1/22:0)+H | CerG1 | 2.051 | <0.001 |
|  |  | Cer(d18:1/24:1)+H | Cer | 2.058 | 0.015 |
|  |  | CerG1(d18:1/22:1)+H | CerG1 | 2.068 | 0.001 |
|  |  | PE(56:2)-H | PE | 2.069 | 0.002 |
|  |  | Cer(d18:1/23:0)+H | Cer | 2.072 | 0.003 |
|  |  | LPE(20:1)-H | LPE | 2.098 | 0.001 |
|  |  | CerG1(d18:1/22:0)+H | CerG1 | 2.114 | 0.002 |
|  |  | PC(24:0/18:2)+HCOO | PC | 2.120 | <0.001 |
|  |  | SM(d18:1/27:0)+H | SM | 2.122 | 0.014 |
|  |  | PI(18:0/20:3)-H | PI | 2.128 | 0.001 |
|  |  | Cer(d32:3)+H | Cer | 2.174 | 0.044 |
|  |  | Cer(d18:0+pO/24:0+O)+HCOO | Cer | 2.193 | 0.012 |
|  |  | Cer(d18:0/24:1)+H | Cer | 2.201 | 0.005 |
|  |  | Cer(d18:1/22:0)+H | Cer | 2.208 | 0.001 |
|  |  | Cer(d18:0/24:0)+H | Cer | 2.228 | 0.001 |
|  |  | PC(18:1p/20:1)+HCOO | PC | 2.236 | <0.001 |
|  |  | PC(37:1p)+H | PC | 2.287 | 0.008 |
|  |  | PI(18:0/18:1)-H | PI | 2.327 | 0.005 |
|  |  | Cer(d18:1/22:1)+H | Cer | 2.343 | 0.002 |
|  |  | PE(18:1p/20:1)+H | PE | 2.364 | <0.001 |
|  |  | PC(18:1/22:6)+HCOO | PC | 2.381 | <0.001 |
|  |  | CerG1(d18:1/24:0+O)+H | CerG1 | 2.391 | 0.004 |
|  |  | WE(21:1)+NH4 | WE | 2.468 | <0.001 |
|  |  | MGMG(16:0)+HCOO | MGMG | 2.542 | 0.011 |
|  |  | WE(23:2)+NH4 | WE | 2.643 | <0.001 |
|  |  | CerG1(d18:1/26:0)+H | CerG1 | 2.691 | 0.029 |
|  |  | PC(38:7)+H | PC | 2.732 | <0.001 |
|  |  | DG(18:0/18:1)+NH4 | DG | 2.887 | <0.001 |
|  |  | PE(40:1e)-H | PE | 3.269 | <0.001 |
|  |  | PS(18:1/24:0)-H | PS | 3.274 | <0.001 |
|  |  | PC(42:1e)+H | PC | 3.471 | <0.001 |
|  |  | LPI(18:1)-H | LPI | 3.883 | <0.001 |
|  |  | PE(34:2p)-H | PE | 4.491 | <0.001 |
|  |  | TG(18:3/18:2/18:2)+NH4 | TG | 4.765 | 0.036 |
|  |  | PC(33:0)+H | PC | 6.113 | <0.001 |
