# Supplementary material for: The Impact of Electroacupuncture Early Intervention on the Brain Lipidome in a Mouse Model of Post-traumatic Stress Disorder
Source: Front Mol Neurosci. 2022 Feb 10;15:812479. doi: 10.3389/fnmol.2022.812479 (PMC8866946; doi:10.3389/fnmol.2022.812479)
Supplement: Supplementary Table S5 — Normalized lipids after EA treatment in the hippocampus and PFC. [file Table_5.DOCX]

**Table S5. Normalized lipids after EA treatment in the hippocampus and PFC**

| **LipidIon** | **Class** | **PTSD+Sham *vs.* Sham** | | **PTSD+EA *vs.* PTSD+Sham** | |
| --- | --- | --- | --- | --- | --- |
|  |  | **Fold change** | ***P* value** | **Fold change** | ***P* value** |
| **Hippocampus** |  |  |  |  |  |
| Cer(d18:0+pO/24:0+O)+HCOO | Cer | 1.526 | 0.008 | 0.635 | 0.004 |
| CerG1(d18:0/24:0+O)+H | CerG1 | 1.591 | 0.001 | 0.492 | < 0.001 |
| CerG1(d18:1/18:1)+H | CerG1 | 1.512 | < 0.001 | 0.621 | < 0.001 |
| CerG1(d18:1/22:1)+H | CerG1 | 1.850 | < 0.001 | 0.611 | 0.002 |
| CerG1(d18:1/24:1)+H | CerG1 | 0.619 | 0.007 | 1.740 | 0.008 |
| CerG1(d18:1/25:0)+H | CerG1 | 1.553 | < 0.001 | 0.572 | < 0.001 |
| CerG1(d58:4)+H | CerG1 | 1.518 | < 0.001 | 0.605 | < 0.001 |
| CL(18:1/16:0/16:0/18:1)-H | CL | 0.582 | 0.001 | 1.576 | 0.003 |
| CL(18:1/18:1/18:1/20:4)-H | CL | 0.644 | < 0.001 | 1.640 | < 0.001 |
| CL(18:2/18:1/16:1/18:1)-H | CL | 0.595 | < 0.001 | 1.691 | < 0.001 |
| CL(18:2/18:1/18:1/18:2)-H | CL | 0.647 | < 0.001 | 1.534 | < 0.001 |
| CL(18:2/20:4/16:0/20:4)-H | CL | 0.263 | < 0.001 | 2.287 | < 0.001 |
| CL(18:2/20:4/16:1/20:4)-H | CL | 0.633 | < 0.001 | 1.625 | < 0.001 |
| CL(18:4/22:6/20:4/18:1)-H | CL | 0.578 | < 0.001 | 1.604 | < 0.001 |
| CL(22:6/18:1/16:0/20:4)-H | CL | 0.422 | < 0.001 | 2.747 | < 0.001 |
| CL(22:6/20:4/22:6/22:6)-H | CL | 0.597 | < 0.001 | 1.889 | < 0.001 |
| CL(22:6/22:6/16:1/20:4)-H | CL | 0.570 | < 0.001 | 1.943 | < 0.001 |
| CL(22:6/22:6/22:6/20:4)-H | CL | 0.560 | < 0.001 | 1.527 | 0.009 |
| Co(Q9)+NH4 | Co | 0.556 | < 0.001 | 1.696 | < 0.001 |
| DG(18:1/22:1)+NH4 | DG | 2.344 | < 0.001 | 0.666 | 0.005 |
| FA(22:6)-H | FA | 0.571 | < 0.001 | 2.859 | < 0.001 |
| LPE(18:0)-H | LPE | 0.598 | < 0.001 | 1.734 | < 0.001 |
| LPS(18:1)-H | LPS | 0.593 | < 0.001 | 1.501 | < 0.001 |
| MGDG(10:4/22:6)+HCOO | MGDG | 0.516 | < 0.001 | 2.819 | < 0.001 |
| MGDG(18:3/18:3)+HCOO | MGDG | 0.590 | < 0.001 | 1.838 | 0.001 |
| PC(26:2p)+H | PC | 0.511 | < 0.001 | 1.753 | < 0.001 |
| PC(34:1)+H | PC | 0.421 | < 0.001 | 1.777 | 0.001 |
| PC(38:2e)+H | PC | 0.560 | < 0.001 | 1.870 | < 0.001 |
| PC(39:0)+H | PC | 0.643 | 0.001 | 1.518 | < 0.001 |
| PC(42:8)+H | PC | 0.622 | < 0.001 | 1.610 | < 0.001 |
| PC(58:6)+H | PC | 0.582 | < 0.001 | 0.652 | < 0.001 |
| PE(18:0/18:1)-H | PE | 1.554 | < 0.001 | 0.626 | < 0.001 |
| PG(39:6)-H | PG | 0.486 | < 0.001 | 1.797 | < 0.001 |
| PS(16:0/18:1)-H | PS | 0.613 | < 0.001 | 1.523 | 0.001 |
| PS(20:4/22:6)-H | PS | 1.583 | < 0.001 | 0.369 | < 0.001 |
| PS(36:3p)-H | PS | 1.521 | < 0.001 | 0.639 | < 0.001 |
| PS(40:6p)-H | PS | 1.544 | < 0.001 | 0.363 | < 0.001 |
| PS(44:11)-H | PS | 1.524 | < 0.001 | 0.433 | < 0.001 |
| SM(d22:1/16:0)+HCOO | SM | 0.511 | < 0.001 | 1.735 | < 0.001 |
| SM(d34:1)+H | SM | 0.538 | < 0.001 | 1.841 | < 0.001 |
| SM(d36:0)+H | SM | 0.578 | 0.001 | 1.523 | 0.005 |
| TG(16:0/18:1/24:0)+NH4 | TG | 1.519 | < 0.001 | 0.666 | < 0.001 |
|  |  |  |  |  |  |
| **Prefrontal cortex** |  |  |  |  |  |
| LPG(16:0)-H | LPG | 0.434 | < 0.001 | 2.901 | < 0.001 |
| DG(16:0/18:2)+NH4 | DG | 0.471 | < 0.001 | 1.776 | 0.032 |
| PS(18:1/18:1)+H | PS | 0.506 | < 0.001 | 1.569 | < 0.001 |
| Cer(d20:1)+H | Cer | 0.544 | < 0.001 | 1.585 | 0.022 |
| LPG(20:4)-H | LPG | 0.549 | < 0.001 | 2.725 | < 0.001 |
| LPC(16:1p)+H | LPC | 0.582 | 0.046 | 1.547 | 0.026 |
| PG(40:5)+NH4 | PG | 0.658 | 0.001 | 1.621 | 0.019 |
| Co(Q9)+NH4 | Co | 0.669 | 0.001 | 2.057 | < 0.001 |
| LPE(20:3)-H | LPE | 2.076 | < 0.001 | 0.330 | < 0.001 |
| LPE(20:2)-H | LPE | 2.242 | 0.001 | 0.351 | < 0.001 |
| DG(32:0e)+Na | DG | 1.681 | 0.011 | 0.643 | 0.029 |
