# Supplementary material for: The Impact of Electroacupuncture Early Intervention on the Brain Lipidome in a Mouse Model of Post-traumatic Stress Disorder
Source: Front Mol Neurosci. 2022 Feb 10;15:812479. doi: 10.3389/fnmol.2022.812479 (PMC8866946; doi:10.3389/fnmol.2022.812479)
Supplement: Supplementary Table S6 — Correlation between PTSD-like behaviors and levels of lipid species in the hippocampus and PFC. [file Table_6.docx]

| **Table S6. Correlation between PTSD-like behaviors and levels of lipid species in the hippocampus and PFC** | | | | | | | | |
| --- | --- | --- | --- | --- | --- | --- | --- | --- |
| LipidIon | Time in center (s) | | Time in open arms (s) | | Contextual freezing time(s) | | Cued freezing time(s) | |
|  | r value | *P* value | r value | *P* value | r value | *P* value | r value | *P* value |
| **Hippocampus** |  |  |  |  |  |  |  |  |
| Cer(d18:0+pO/24:0+O)+HCOO | -0.124 | 0.499 | -0.303 | < 0.001 | 0.511 | 0.003 | 0.581 | < 0.001 |
| CerG1(d18:0/24:0+O)+H | -0.417 | 0.017 | -0.47 | < 0.001 | 0.379 | 0.032 | 0.504 | < 0.001 |
| CerG1(d18:1/18:1)+H | -0.439 | 0.012 | -0.289 | < 0.001 | 0.488 | 0.005 | 0.483 | < 0.001 |
| CerG1(d18:1/22:1)+H | -0.36 | 0.043 | -0.425 | < 0.001 | 0.414 | 0.019 | 0.505 | < 0.001 |
| CerG1(d18:1/24:1)+H | 0.383 | 0.03 | 0.388 | < 0.001 | -0.37 | 0.037 | -0.382 | < 0.001 |
| CerG1(d18:1/25:0)+H | -0.376 | 0.034 | -0.316 | < 0.001 | 0.398 | 0.024 | 0.444 | < 0.001 |
| CerG1(d58:4)+H | -0.39 | 0.027 | -0.412 | < 0.001 | 0.471 | 0.007 | 0.41 | < 0.001 |
| CL(18:1/16:0/16:0/18:1)-H | 0.462 | 0.008 | 0.394 | < 0.001 | -0.482 | 0.005 | -0.628 | < 0.001 |
| CL(18:1/18:1/18:1/20:4)-H | 0.619 | < 0.0001 | 0.546 | < 0.001 | -0.547 | 0.001 | -0.479 | < 0.001 |
| CL(18:2/18:1/16:1/18:1)-H | 0.426 | 0.015 | 0.669 | < 0.001 | -0.457 | 0.009 | -0.412 | < 0.001 |
| CL(18:2/18:1/18:1/18:2)-H | 0.607 | < 0.0001 | 0.616 | < 0.001 | -0.519 | 0.002 | -0.509 | < 0.001 |
| CL(18:2/20:4/16:0/20:4)-H | 0.445 | 0.011 | 0.452 | < 0.001 | -0.495 | 0.004 | -0.592 | < 0.001 |
| CL(18:2/20:4/16:1/20:4)-H | 0.592 | < 0.0001 | 0.637 | < 0.001 | -0.462 | 0.008 | -0.311 | < 0.001 |
| CL(18:4/22:6/20:4/18:1)-H | 0.494 | 0.004 | 0.693 | < 0.001 | -0.373 | 0.036 | -0.405 | < 0.001 |
| CL(22:6/18:1/16:0/20:4)-H | 0.503 | 0.003 | 0.587 | < 0.001 | -0.48 | 0.005 | -0.481 | < 0.001 |
| CL(22:6/22:6/22:6/20:4)-H | 0.103 | 0.574 | 0.046 | < 0.001 | -0.353 | 0.048 | -0.273 | < 0.001 |
| Co(Q9)+NH4 | 0.456 | 0.009 | 0.534 | < 0.001 | -0.633 | < 0.001 | -0.686 | < 0.001 |
| DG(18:1/22:1)+NH4 | -0.221 | 0.223 | 0.048 | < 0.001 | 0.18 | 0.324 | 0.147 | < 0.001 |
| FA(22:6)-H | 0.357 | 0.045 | 0.297 | < 0.001 | -0.322 | 0.073 | -0.247 | < 0.001 |
| LPE(18:0)-H | 0.506 | 0.003 | 0.495 | < 0.001 | -0.604 | < 0.001 | -0.604 | < 0.001 |
| LPS(18:1)-H | 0.167 | 0.36 | 0.041 | < 0.001 | -0.267 | 0.140 | -0.395 | < 0.001 |
| MGDG(10:4/22:6)+HCOO | 0.442 | 0.011 | 0.443 | < 0.001 | -0.415 | 0.018 | -0.411 | < 0.001 |
| MGDG(18:3/18:3)+HCOO | 0.455 | 0.009 | 0.29 | < 0.001 | -0.464 | 0.008 | -0.522 | < 0.001 |
| PC(26:2p)+H | 0.301 | 0.095 | 0.544 | < 0.001 | -0.305 | 0.09 | -0.318 | < 0.001 |
| PC(34:1)+H | 0.475 | 0.006 | 0.232 | < 0.001 | -0.481 | 0.005 | -0.42 | < 0.001 |
| PC(38:2e)+H | 0.522 | 0.002 | 0.543 | < 0.001 | -0.493 | 0.004 | -0.606 | < 0.001 |
| PC(39:0)+H | 0.346 | 0.052 | 0.531 | < 0.001 | -0.345 | 0.053 | -0.362 | < 0.001 |
| PC(42:8)+H | 0.502 | 0.003 | 0.537 | < 0.001 | -0.592 | < 0.001 | -0.56 | < 0.001 |
| PC(58:6)+H | 0.422 | 0.016 | 0.462 | < 0.001 | -0.489 | 0.004 | -0.436 | < 0.001 |
| PE(18:0/18:1)-H | -0.548 | 0.001 | -0.497 | < 0.001 | 0.621 | < 0.001 | 0.672 | < 0.001 |
| PG(39:6)-H | 0.352 | 0.048 | 0.565 | < 0.001 | -0.622 | < 0.001 | -0.64 | < 0.001 |
| PS(16:0/18:1)-H | 0.32 | 0.074 | 0.332 | < 0.001 | -0.568 | 0.001 | -0.519 | < 0.001 |
| PS(20:4/22:6)-H | -0.461 | 0.008 | -0.458 | < 0.001 | 0.437 | 0.012 | 0.359 | < 0.001 |
| PS(36:3p)-H | -0.078 | 0.671 | 0.102 | < 0.001 | 0.196 | 0.281 | 0.144 | < 0.001 |
| PS(44:11)-H | -0.472 | 0.006 | -0.403 | < 0.001 | 0.51 | 0.003 | 0.458 | < 0.001 |
| PS(40:6p)-H | -0.467 | 0.007 | -0.515 | < 0.001 | 0.47 | 0.007 | 0.426 | < 0.001 |
| SM(d22:1/16:0)+HCOO | 0.527 | 0.002 | 0.541 | < 0.001 | -0.611 | < 0.001 | -0.526 | < 0.001 |
| SM(d34:1)+H | 0.365 | 0.04 | 0.342 | < 0.001 | -0.546 | 0.001 | -0.501 | < 0.001 |
| SM(d36:0)+H | 0.381 | 0.031 | 0.329 | < 0.001 | -0.459 | 0.008 | -0.323 | < 0.001 |
| TG(16:0/18:1/24:0)+NH4 | -0.364 | 0.04 | -0.36 | < 0.001 | 0.344 | 0.054 | 0.547 | < 0.001 |
| CL(22:6/20:4/22:6/22:6)-H | 0.416 | 0.018 | 0.386 | < 0.001 | -0.534 | 0.002 | -0.357 | < 0.001 |
| CL(22:6/22:6/16:1/20:4)-H | 0.307 | 0.087 | 0.135 | < 0.001 | -0.359 | 0.044 | -0.319 | < 0.001 |
|  |  |  |  |  |  |  |  |  |
| **Prefrontal cortex** |  |  |  |  |  |  |  |  |
| LPG(16:0)-H | 0.315 | 0.079 | 0.208 | 0.253 | -0.472 | 0.006 | -0.396 | 0.025 |
| DG(16:0/18:2)+NH4 | 0.223 | 0.22 | 0.51 | 0.003 | -0.515 | 0.003 | -0.421 | 0.016 |
| PS(18:1/18:1)+H | 0.329 | 0.066 | 0.473 | 0.006 | -0.636 | < 0.001 | -0.698 | < 0.001 |
| Cer(d20:1)+H | 0.368 | 0.038 | 0.228 | 0.209 | -0.167 | 0.361 | -0.226 | 0.215 |
| LPG(20:4)-H | 0.348 | 0.051 | 0.391 | 0.027 | -0.482 | 0.005 | -0.389 | 0.028 |
| LPC(16:1p)+H | 0.176 | 0.336 | 0.319 | 0.075 | -0.391 | 0.027 | -0.385 | 0.03 |
| PG(40:5)+NH4 | 0.293 | 0.103 | 0.295 | 0.101 | -0.385 | 0.029 | -0.426 | 0.015 |
| Co(Q9)+NH4 | 0.325 | 0.069 | 0.299 | 0.096 | -0.466 | 0.007 | -0.289 | 0.109 |
| LPE(20:3)-H | -0.243 | 0.18 | -0.279 | 0.121 | 0.456 | 0.009 | 0.479 | 0.006 |
| LPE(20:2)-H | -0.201 | 0.269 | -0.232 | 0.201 | 0.388 | 0.028 | 0.429 | 0.014 |
| DG(32:0e)+Na | -0.508 | 0.754 | -0.355 | 0.046 | 0.633 | < 0.001 | 0.673 | < 0.001 |
